# A Comparison of Aggregate P-Value Methods and Multivariate Statistics for Self-Contained Tests of Metabolic Pathway Analysis

**DOI:** 10.1371/journal.pone.0125081

**Published:** 2015-04-30

**Authors:** Matthew W. Mitchell

**Affiliations:** Metabolon, Inc., Durham, NC, United States of America; Wayne State University, UNITED STATES

## Abstract

For pathway analysis of genomic data, the most common methods involve combining p-values from individual statistical tests. However, there are several multivariate statistical methods that can be used to test whether a pathway has changed. Because of the large number of variables and pathway sizes in genomics data, some of these statistics cannot be computed. However, in metabolomics data, the number of variables and pathway sizes are typically much smaller, making such computations feasible. Of particular interest is being able to detect changes in pathways that may not be detected for the individual variables. We compare the performance of both the p-value methods and multivariate statistics for self-contained tests with an extensive simulation study and a human metabolomics study. Permutation tests, rather than asymptotic results are used to assess the statistical significance of the pathways. Furthermore, both one and two-sided alternatives hypotheses are examined. From the human metabolomic study, many pathways were statistically significant, although the majority of the individual variables in the pathway were not. Overall, the p-value methods perform at least as well as the multivariate statistics for these scenarios.

## Introduction

In metabolomics, we are not only interested in changes for individual metabolites, but also groups of related metabolites, which may be metabolites in the same class, metabolites in the same pathway, or other groups of bio-signatures. For simplicity, henceforth, all these categories will be referred to as “pathways.” In particular we are interested in the cases where the individual metabolites miss the cut-off for statistical significance from univariate analyses, but in aggregate are found to be statistically significant. For example, suppose we observe eight metabolites in a pathway with p-values of 0.07. If the pair-wise correlations (unless otherwise stated, “correlation” refers to the Pearson correlation) are 0.99, then we expect the aggregate p-value to be similar to an individual p-value. However, if these are statistically independent, then the Fisher meta-analysis [1] p-value = 0.0003. So the aggregate p-value could range from 0.07 (all correlated = 1) to 0.0003. Hence, it is desirable to formally test whether a pathway is changed.

In genomics, there are two categories of pathway analysis: (1) competitive tests and (2) self-contained tests [2, 3]. Competitive tests assess whether there are more changes for those genes in a given pathway than those not in that pathway. Hence, the scenario in the previous paragraph would not be detected if a p-value < 0.05 is defined as a metabolite “changing.” The self-contained tests assess whether the pathway has changed without regard to genes in other pathways. These tests are of more interest for our applications and will be the focus of this paper.

For genomics pathway analysis, the methods for self-contained tests often involve combining the p-values, and in some cases “data modeling” (e.g., logistic regression, principal components analysis) [2–5] is performed, but various multivariate statistics, such as Hotelling’s *T* ^2^ statistic [6], are not considered. Conversely, in the literature of multivariate methods, the aforementioned methods are not considered, but instead multivariate statistics are employed [7–14]. Some of these methods, such as Hotelling’s *T* ^2^ statistic, require the inversion of the sample covariance matrix, which is not possible when the number of observations is less than the number of variables, as is typically the case for-*omics* data. However, other multivariate statistics such as those proposed by Dempster [7, 8], Bai and Saranadasa [9], and Srivastava and Du [10] do not require the inversion of the sample covariance matrix. The distributions of some of these statistics, such as the Bai-Saranadasa statistic [9] rely on asymptotic results, which require even larger sample sizes. Thus, in genomics, many of these statistics will not apply. However, while genomics data sets typically have over 10,000 variables, metabolomics sets often have fewer than 1,000 (chemo-centric, rather than ion-centric [15]). Furthermore, the sizes of the pathways for metabolomics are also significantly smaller, with many containing fewer than 20 metabolites. Thus, these multivariate statistics can apply in many cases for metabolomics data.

In the next section, we describe various methods that aggregate p-values and methods that employ multivariate statistics. Only one “data modeling” approach is considered because for the global models mentioned in [3], logistic regression performed on multiple principal components often failed to converge for the smaller sample sizes in the simulation study. Many of the tests described are two-sided tests; however, we often have pre-specified alternatives, i.e., we expect that the metabolites in the pathway to change in a certain direction, so statistics that can take these into account are also desirable. We thus adapt several methods for one-sided alternatives. These statistical tests are compared in a simulation study and are then compared in an application to a human metabolomics study.

## Materials and Methods

Let **X**_11_, **X**_12_, …, **X**_1,n1_ be random vectors from a distribution with mean **μ**_1_ = (μ_11_, μ _21_,.., μ_m1_), and let **X**_12_, **X**_22_, …, **X**_2,n2_ be random vectors from a distribution with mean **μ**_2_ = (μ_12_, μ_22_,.., μ_m2_). Then, the hypotheses of interest are H_0_: **μ**_1_ = **μ**_2_ vs. H_a_: **μ**_1_ ≠ **μ**_2_. Here *n*_1_ and *n*_2_ represent the two sample sizes, and *m* represents the number of variables. Hence, we are testing whether no metabolites are truly differential against the alternative that at least one metabolite is truly differential.

### Test Statistics for Pathway Analysis

The following are compared for testing the above hypothesis.

1. **Fisher’s method.** Fisher’s method [1] is often used in meta-analysis for testing the same hypothesis across different data sets. So in this context, each member of the pathway is seen as a representative of the same biology. Hence, this test will not work well for the case where one variable changes, but the others do not. However, this is not of primary interest for our applications. Let *p*_i_, *i* = 1,2, …, *m*, be the set of p-values for the *m* members of the pathway. For example, these could be the p-values that result from performing two-sided t-tests for each metabolite. If the variables are independent, then the p-values have a Uniform(0,1) distribution, and thus the statistic $X=-2\sum_{i=1}^{m}\text{log}\;\left(p_{i}\right)$ has a chi-squared distribution with 2*m degrees of freedom. Since, in the scenarios examined, we are considering related metabolites, which often have strong correlations, we use a permutation test as described by Fridley [3] instead of the chi-squared test.
2. **Tail Strength.** The tail strength measure [4] was developed to give an overall measure of significance for a set of hypotheses and has also been used to test significance of pathways [2, 3]. The test statistic compares the ordered p-values to the expected moments of the Uniform(0,1) distribution, i.e., $TS=\frac{1}{m}\sum_{i=1}^{m}\left(\frac{i-p_{\left(i\right)}(m+1)}{i}\right)$, where *p*_(i)_ is the *i*th ordered p-value, i.e., *p*_(1)_ is the smallest p-value and *p*_(m)_ is the largest p-value. This test is approximately normal when *m* is large, which is not the case in our scenarios, so as with (1) we determine the distribution with a permutation test. Alternatively, since the variables are correlated, the moments could also be estimated from the permutation distribution, but this was not done here.
3. **Adaptive Rank Truncated Product.** For the adaptive rank truncated product method (ARTP) [5], the *J* smallest p-values are used for the statistic *W*_*J*_ = *p*_(1)_ *p*_(2)_…*p*_(*J*)_, where *J* is chosen to minimize the p-value. More specifically, first permute the group labels *B* times in order to generate *B* data sets. Next, for each of these data sets compute the *m* p-values, *p* ^b^_(1)_, *p* ^b^_(2)_,…, *p* ^b^_(m)_. Then for each truncation point *J*, 1 ≤ *J* ≤ *m*, and each data set *b*, 0 ≤*b* ≤ *B* (*b* = 0 corresponds to the original data set), compute $W_{J}^{b}=\prod_{i=1}^{J}p_{\left(i\right)}^{b}$. Now for each *b* and *J*, compute its estimated p-value with Ge’s algorithm $q_{J}^{b}=\frac{\sum_{b*=0}^{B}I\left(W_{J}^{b*}\leq W_{J}^{b}\right)}{B+1}$, where *I* is the usual indicator function. Next, let $MinP^{b}=\text{min}\;q_{J}^{b}$ over 1 ≤ *J* ≤ *m*. Then the p-value for the adaptive rank truncated product is given as $\frac{\sum_{b=0}^{B}I\left(MinP^{b}\leq MinP^{0}\right)}{B+1}$.
4. **Principal Components Analysis (PCA).** There are various ways a principal components analysis (PCA) can be performed—one may simply choose a set number of components or choose the first *k* components so that a certain percentage of the total variation of is accounted for (such as 80%) [3], and then perform logistic regression on these components. Because the numbers of variables in the pathways under consideration are low, we simply choose the first component, and then perform a two-sample t-test. One advantage of this method is that a single score can be obtained for each sample and fold changes computed.
5. **Hotelling’s T** ^**2**^. Let **X**_11_, **X**_12_, …, **X**_1,n1_ be i.i.d. random vectors from a multinormal distribution with mean **μ**_1_ = (μ _11_, μ _21_,.., μ _m1_) and covariance matrix **∑**, and let **X**_12_, **X**_22_, …, **X**_2,n2_ be random vectors from a multinormal distribution with mean **μ**_2_ = (μ _12_, μ _22_,.., μ _m2_) and covariance matrix **∑**. Then under the null hypothesis, $\left(\frac{n_{1}+n_{2}-m-1}{\left(n_{1}+n_{2}-2\right)m}\right)t^{2}∼F\left(m,n_{1}+n_{2}-1-m\right)$ where $t^{2}=\left(\frac{n_{1}n_{2}}{n_{1}+n_{2}}\right){\left({\bar{X}}_{1}-{\bar{X}}_{2}\right)}^{'}\;S_{p}^{-1}\;\left({\bar{X}}_{1}-{\bar{X}}_{2}\right)$ with ${\bar{X}}_{1}$ and ${\bar{X}}_{2}$ representing the usual vector of sample means and ***S***_p_ representing the pooled estimate of the covariance matrix, i.e., $S_{p}=\frac{\left(n_{1}-1\right)S_{1}+\left(n_{2}-1\right)S_{2}}{n_{1}+n_{2}-2}$, with ***S***_1_ and ***S***_2_ representing the usual sample covariance matrices [6]. As one can see, this requires multivariate normality, the same covariance matrix for each group, and requires that the total number of observations (*n*_1_ + *n*_2_) is greater than (*m*+1). If the normality assumption is questionable, a permutation distribution could be used instead of the F-statistic.
6. **Dempster’s Test.** Dempster also developed multivariate statistics for these scenarios [7, 8]. This test statistic is given by $DM=\frac{\frac{n_{1}n_{2}}{n_{1}+n_{2}}{\left({\bar{X}}_{1}-{\bar{X}}_{2}\right)}^{'}\;\left({\bar{X}}_{1}-{\bar{X}}_{2}\right)}{\text{tr}\left(S_{p}\right)}$, where *tr* represents the trace function. Let *n* = *n*_1_ + *n*_2_–2. When the data are sampled from a multivariate normal distribution, this has an approximate *F*-distribution with *r* and *nr* degrees of freedom, where *r* is given by $r=\frac{{\left(\text{tr}\;\left(\Sigma \right)\right)}^{2}}{\text{tr}\;\Sigma^{2}}$ [9,10] and can be estimated by $\hat{r}=m\frac{{\hat{a}}_{1}^{2}}{{\hat{a}}_{2}}$ with ${\hat{a}}_{1}=\frac{\text{tr}\;\left(S_{p}\right)}{m}$ and ${\hat{a}}_{2}=\frac{n^{2}}{\left(n-1\right)\left(n-2\right)}\frac{1}{m}\left[\text{tr}(S_{p}^{2})-\frac{{\left(\text{tr}\;\left(S_{p}\right)\right)}^{2}}{n}\right]\left[10\right].$ Rather than using the approximation for the test statistic’s distribution, which relies on both the *F*-approximation and the approximation for the degrees of freedom, a permutation based value is used instead. Like Hotelling’s *T* ^2^, this requires the same covariance matrix for each group, but this has a strong advantage over Hotelling’s *T* ^2^ because this test statistic does not require the inversion of the sample covariance matrix.
7. **Bai-Saranadasa Test.** Bai and Saranadasa [9] also developed a test statistic that does not require the inversion of the sample covariance matrix. Let *n* = *n*_1_ + *n*_2._ − 2. Their test statistic is given by $BS=\frac{\frac{n_{1}n_{2}}{n_{1}+n_{2}}{\left({\bar{X}}_{1}-{\bar{X}}_{2}\right)}^{'}\left({\bar{X}}_{1}-{\bar{X}}_{2}\right)-\text{tr}S_{p}}{\sqrt{\frac{2\left(n+1\right)}{n}}\;B_{n}}$, where $B_{n}^{2}=\frac{n^{2}}{\left(n+2\right)\left(n-1\right)}\left(\text{tr}S_{p}^{2}-\frac{1}{n}{\left(\text{tr}S_{p}\right)}^{2}\right)$. This statistic has an asymptotic *N*(0,1) distribution, but since the sample sizes may be too small, we again use a permutation distribution of the statistic to determine the p-value.
8. **Srivastava-Du Test.** Srivastava and Du [10] also developed a multivariate test statistic that does not require the inversion of the sample covariance matrix. This test statistic is given by $SD=\frac{\frac{n_{1}n_{2}}{n_{1}+n_{2}}{\left({\bar{X}}_{1}-{\bar{X}}_{2}\right)}^{'}\;D_{S}^{-1}\;\left({\bar{X}}_{1}-{\bar{X}}_{2}\right)-\frac{nm}{n-2}}{\sqrt{2\left(\text{tr}(R^{2})-\frac{m^{2}}{n}\right)c_{m,n}}}$, where **D**_**s**_ is the matrix consisting of the sample pooled variances on the diagonal and zero otherwise, *c*_*m*,*n*_ = 1 + tr(**R** ^2^)/*m* ^3/2^, and **R** is the matrix obtained from $D_{s}^{-1/2}\;S_{p}\;D_{S}^{-1/2}$, where **D**_s_^-1/2^ is the matrix with the reciprocals of the pooled standard deviations along the diagonal and zero otherwise. Similar to (1)-(3), (6), (7), a permutation distribution is used to compute the p-values.
9. For one-sided alternatives, we propose a simple modification of statistics of the form (2.4) given in Sen [11], which was used in the context of union-intersection tests for one-sample problems: $T_{n}\left(b\right)=\frac{\sqrt{n}b^{\prime }\bar{X}}{\sqrt{b^{\prime }Sb}}$ where $\bar{X}$ and **S** are the usual one-sample mean vector and covariance matrix for testing a series of hypotheses of the form *H*_0,***b***_ = ***b′*** *θ =* 0. We propose the natural two-sample analog. Let **a** represent the vector of coefficients related to the hypothesized direction of change. For example, if one is considering four variables and expects them to increase under one experimental condition (i.e., one expects that the means for group 2 are greater than those for group 1), then **a** = (1,1,1,1); or if we expect the first two variables to increase, but the second two variables to decrease then **a** = (1,1,-1,-1). The proposed statistic for one-sided alternatives, is $T_{0}=\frac{a'\left({\bar{X}}_{1}-{\bar{X}}_{2}\right)\sqrt{\frac{n_{1}n_{2}}{n_{1}+n_{2}}}}{\sqrt{a'S_{p}a}}$. As with (6)-(8), this does not require inversion of the sample covariance matrix. Furthermore, we are not concerned with the asymptotic distribution, as a permutation distribution will be used.
10. We also consider (1)-(3) by computing p-values for the one-sided tests. For example, if the individual two-sided p-values for each variable from a t-test are computed to determine the statistics in (1)-(3), then here, the one-sided p-values are computed to determine the statistics in (1)-(3).
11. **Additional one-sided alternatives.** In the literature there are several publications concerning testing whether the parameters fall in the positive orthant, i.e., where at least one of the mean differences is positive, and the others are non-negative [12–14]. The common form of testing this is by using the test statistic in conjunction with the condition that the sum of the sample mean differences is positive: for a level of 0.05, reject if the test statistic has p-value less than 0.10 and the sum of the mean differences is positive [12].

### Data

#### Simulation Study

For the simulation study, we use sample sizes and variable sizes often seen in metabolomic animal studies, which typically have low numbers of animals used (of the 100 most recently analyzed data sets at Metabolon, 39 were rodent studies with median group size = 5, and 38 of the 39 had group sizes from 2 to 8 animals) and small pathway sizes. We use *n*_1_ = *n*_2_ = 5, *n*_1_ = *n*_2_ = 10, and *m* = 4, 8. The data are simulated from multinormal distributions with the same covariance matrix for each group. The correlation matrices are of the form where all the off-diagonal elements are equal to ρ. Let **R**_1_, **R**_2_, **R**_3_, **R**_4_, represent the correlation matrices where ρ = 0, 0.5, 0.7, and 0.9, respectively. Let **1**_k_ and **0**_k_ represent the vectors of length *k* (1, 1, …, 1)_k_ and (0, 0, …, 0)_k_, respectively. The population standard deviations are **σ**_11_ = 0.3*I_4_, **σ**_12_ = (0.15, 0.25, 0.35, 0.45), **σ**_21_ = 0.3*I_8_, and **σ**_22_ = (**σ**_12_, **σ**_12_) = (0.15, 0.25, 0.35, 0.45, 0.15, 0.25, 0.35, 0.45). The differences in means assessed are **m**_10_ = **0**_4_, **m**_11_ = 0.15* **I**_4_, **m**_12_ = 0.3* **I**_4_, **m**_13_ = (0.3,0,0,0), **m**_20_ = **0**_8_, **m**_21_ = 0.15*I_8_, **m**_22_ = 0.3* **I**_8_, and **m**_23_ = (0.3,0,0,0,0,0,0,0).

For each permutation test, 1,000 permutations were used to determine the p-value, i.e., the group labels were permuted 1,000 times, and then the test statistic was computed for each permuted data set. The p-value is computed as the proportion of these values at least as large as the observed value. More formally, let *Y*_0_ represent the test statistic (from (1)–(13)), and let *Y*_1_, *Y*_2_…*Y*_1000_ represent the test statistics generated from the permuted data sets. Then $p=\left(\frac{1}{1000}\right)\sum_{k=1}^{1000}I\left(Y_{k}\geq Y_{0}\right)$, where *p* represents the empirical p-value. Next 1,000 simulations runs were performed to determine the empirical type I error and power for the combination of parameters under consideration, i.e., the power or type I error is equal to $\left(\frac{1}{1000}\right)\sum_{j=1}^{1000}I(p_{j}<\alpha )$, where *α* is the level of the test and *p*_j_ is the p-value computed from the *j* ^th^ simulation run. For the one-sided alternatives, the tests used **a** = **I**_4_ or **a** = **I**_**8**_ (i.e., all mean changes are positive). All simulations were performed in *R* [16] and the computation of Hotelling’s *T* ^2^ was performed with the package “Hotelling” [17].

#### Insulin Resistance Human Metabolomic Study

Next, we apply these methods to a large human metabolomics data set concerning insulin resistance [18], which is a data set that has been previously published in this journal. Here the comparison is for insulin resistant subjects,” IR”, (n_1_ = 138), vs. insulin sensitive subjects,” IS”, (n_2_ = 261) as defined in the paper. There are many more subjects in this study than many metabolomics data sets, as this was used for human biomarker discovery. More details on the data processing are given in that paper. All statistics are performed on the log-transformed data.

This data set shows many of the difficulties in performing metabolic pathway analysis in practice, many of which are similar problems encountered in genomics pathway analysis. For example, as genes belong to multiple pathways [19, 20], some metabolites belong to multiple pathways. For example, using KEGG [21], alanine is listed in 17 pathways, many of which overlap. Furthermore, incomplete, limited, or erroneous annotations and information poses problems in classifying genes into pathways [19, 20], which also occurs in metabolomics. For example, using KEGG [21] some metabolites are not assigned to any pathways, such as 1-methyladenosine, which has one reaction listed, but no pathways listed. Additionally, in metabolomics, every element in the pathway may not be observed depending on the concentration of the metabolite and the technology used to measure its levels. Note that as the statistics used are functional class scoring methods [19], any specific relationships within a pathway are not modeled [19], and the analyses performed do not take into account relationships of the pathways to each other [19, 20].

For this data set, each metabolite was assigned to a single pathway as defined by in-house experts, who made use of such public databases such as KEGG [21]. As mentioned previously, “pathway” is being used to refer to any group of related metabolites, so metabolites in the same class may be grouped, even though they do not belong the same physical pathway. The pathways are larger in genomics studies where the minimum and maximum numbers of elements in the pathways considered are often 10 and 100–200 genes, respectively [20]. However, virtually none of the metabolic pathways would satisfy these criteria, as the metabolic pathways tend to be much smaller. Here, pathways with only one representative were excluded from the analysis. Since this data set had large sample sizes, the permutation distributions for each statistic were determined from 10,000 permutations, rather than 1,000 permutations, which were used for the simulation study.

## Results

### Simulation Study

For all tables, MU is the mean difference, σ the vector of the standard deviations, ρ, the pair-wise correlations, *N* = *n*_1_ = *n*_2_, FX = Fisher’s statistic using the chi-squared distribution, FP = Fisher’s statistic using the permutation distribution, TS = tail strength statistic, ARTP = adaptive rank truncated product, PCA, the results from performing the two-sample t-test on the first principal component, HT = Hotelling’s’ *T* ^2^, BSN = Bai-Saranadasa statistic using the normal approximation, BSP = Bai-Saranadasa statistic using the permutation distribution, DM = Dempster’s statistic, SD = Srivastava and Du’s statistic and T_0_ = the two-sample analog of Sen’s statistic given in (9).

We first assess the type I error for each of the tests under the various parameter combinations listed in the previous section. The chi-squared statistic for (1) (FX) and the normal approximation for (7) (BSN) are also examined in order to see how close these are to the desired type I error. A level of α = 0.05 was used for each test. As we can see from Tables 1 and 2, the non-permutation based measurements (FX, BSN) have type I errors larger than the nominal levels, especially with highly correlated variables, as expected. All the others (the permutation-based measures) have type I errors at the nominal level. The results for the one-sided tests (not shown) were similar. Note that If the true type I error = 0.05, then the standard error of its estimate is $\sqrt{\frac{0.05*0.95}{1000}}$, which is approximately 0.007.

**Table 1:** Empirical Type 1 Error, 4 variables, two-sided tests.

σ	ρ	N	FX	FP	TS	ARTP	PCA	HT	BSN	BSP	DM	SD
σ11	0.9	5	0.136	0.061	0.061	0.061	0.046	0.052	0.116	0.059	0.059	0.059
σ12	0.9	5	0.141	0.059	0.06	0.062	0.045	0.057	0.12	0.058	0.058	0.06
σ11	0.7	5	0.098	0.051	0.049	0.055	0.043	0.036	0.099	0.039	0.039	0.039
σ12	0.7	5	0.104	0.052	0.052	0.047	0.034	0.048	0.105	0.06	0.06	0.057
σ11	0.5	5	0.099	0.065	0.072	0.062	0.054	0.059	0.123	0.059	0.061	0.064
σ12	0.5	5	0.072	0.042	0.046	0.045	0.032	0.044	0.107	0.055	0.054	0.044
σ11	0	5	0.051	0.055	0.054	0.055	0.051	0.041	0.096	0.053	0.048	0.052
σ12	0	5	0.039	0.045	0.046	0.058	0.034	0.044	0.097	0.059	0.056	0.039
σ11	0.9	10	0.111	0.041	0.04	0.044	0.038	0.04	0.078	0.044	0.044	0.045
σ12	0.9	10	0.127	0.043	0.044	0.043	0.042	0.052	0.08	0.043	0.043	0.044
σ11	0.7	10	0.115	0.052	0.048	0.054	0.048	0.048	0.091	0.037	0.036	0.036
σ12	0.7	10	0.103	0.043	0.042	0.05	0.041	0.043	0.083	0.036	0.036	0.036
σ11	0.5	10	0.095	0.059	0.056	0.064	0.049	0.047	0.093	0.054	0.053	0.051
σ12	0.5	10	0.067	0.045	0.048	0.044	0.041	0.051	0.078	0.053	0.052	0.048
σ11	0	10	0.05	0.051	0.049	0.049	0.044	0.041	0.08	0.035	0.036	0.041
σ12	0	10	0.066	0.065	0.05	0.064	0.055	0.052	0.08	0.055	0.057	0.045

**Table 2:** Empirical Type I, 8 variables, two-sided tests.

σ	ρ	N	FX	FP	TS	ARTP	PCA	HT	BSN	BSP	DM	SD
σ21	0.9	5	0.212	0.056	0.057	0.068	0.05	0.059	0.132	0.049	0.049	0.049
σ22	0.9	5	0.194	0.057	0.057	0.058	0.045	0.045	0.12	0.048	0.048	0.047
σ21	0.7	5	0.148	0.051	0.052	0.056	0.047	0.057	0.111	0.046	0.045	0.049
σ22	0.7	5	0.15	0.052	0.049	0.053	0.047	0.058	0.107	0.044	0.046	0.049
σ21	0.5	5	0.101	0.038	0.042	0.044	0.033	0.05	0.089	0.05	0.049	0.05
σ22	0.5	5	0.14	0.063	0.058	0.064	0.048	0.045	0.107	0.052	0.049	0.051
σ21	0	5	0.053	0.061	0.05	0.064	0.058	0.05	0.097	0.056	0.053	0.057
σ22	0	5	0.039	0.046	0.044	0.052	0.043	0.049	0.084	0.041	0.039	0.063
σ21	0.9	10	0.202	0.06	0.06	0.055	0.059	0.043	0.094	0.045	0.045	0.045
σ22	0.7	10	0.157	0.053	0.059	0.054	0.052	0.058	0.094	0.051	0.051	0.053
σ21	0.5	10	0.132	0.057	0.053	0.054	0.051	0.056	0.1	0.05	0.049	0.05
σ22	0	10	0.049	0.047	0.046	0.047	0.049	0.045	0.077	0.048	0.047	0.051
σ21	0.9	10	0.166	0.051	0.051	0.054	0.049	0.052	0.088	0.05	0.051	0.05
σ22	0.7	10	0.136	0.044	0.045	0.047	0.044	0.058	0.078	0.043	0.045	0.039
σ21	0.5	10	0.121	0.05	0.052	0.051	0.047	0.046	0.08	0.049	0.048	0.045
σ22	0	10	0.048	0.043	0.048	0.054	0.052	0.038	0.083	0.045	0.043	0.044

**Fig 1:**
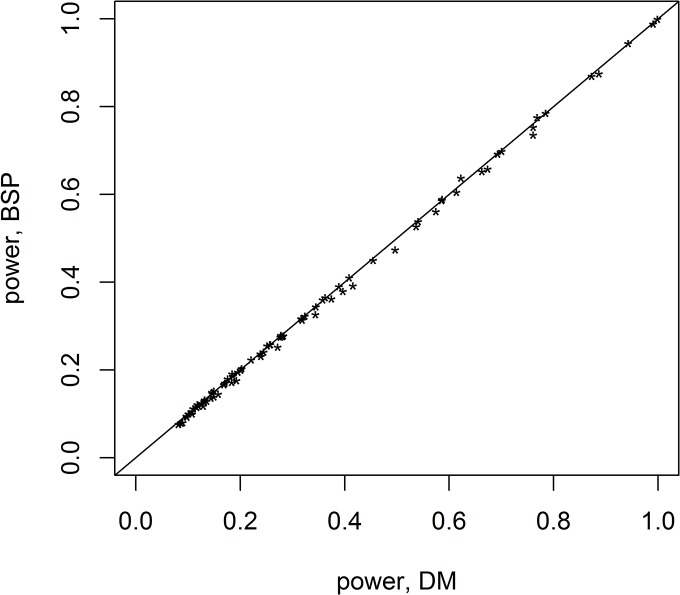
Comparison of the empirical power for Dempster’s test (DM) and Bai-Saranadasa (BSP) for all of the two-sided tests. The line y = x is plotted for comparison.

Because of the inflated type I errors, FX and BSN are not considered, henceforth. The empirical power is computed for the combinations of means and covariances defined in the previous section. Both Bai and Saranadasa [9] and Srivastava and Du [10] have shown that asymptotically, Dempster’s statistic has the same power as the Bai-Saranadasa statistic. For the small sample sizes used in the simulation runs, these two statistics also have essentially equivalent performance as seen in Fig 1, which plots the empirical powers of one against the other (the line y = x is shown for comparison) for all the combinations shown in S1 and S2 Tables (the two-sided tests). Since these two tests are essentially equivalent, the remainder of comparisons with these two will reference only the BSP values.

**Fig 2:**
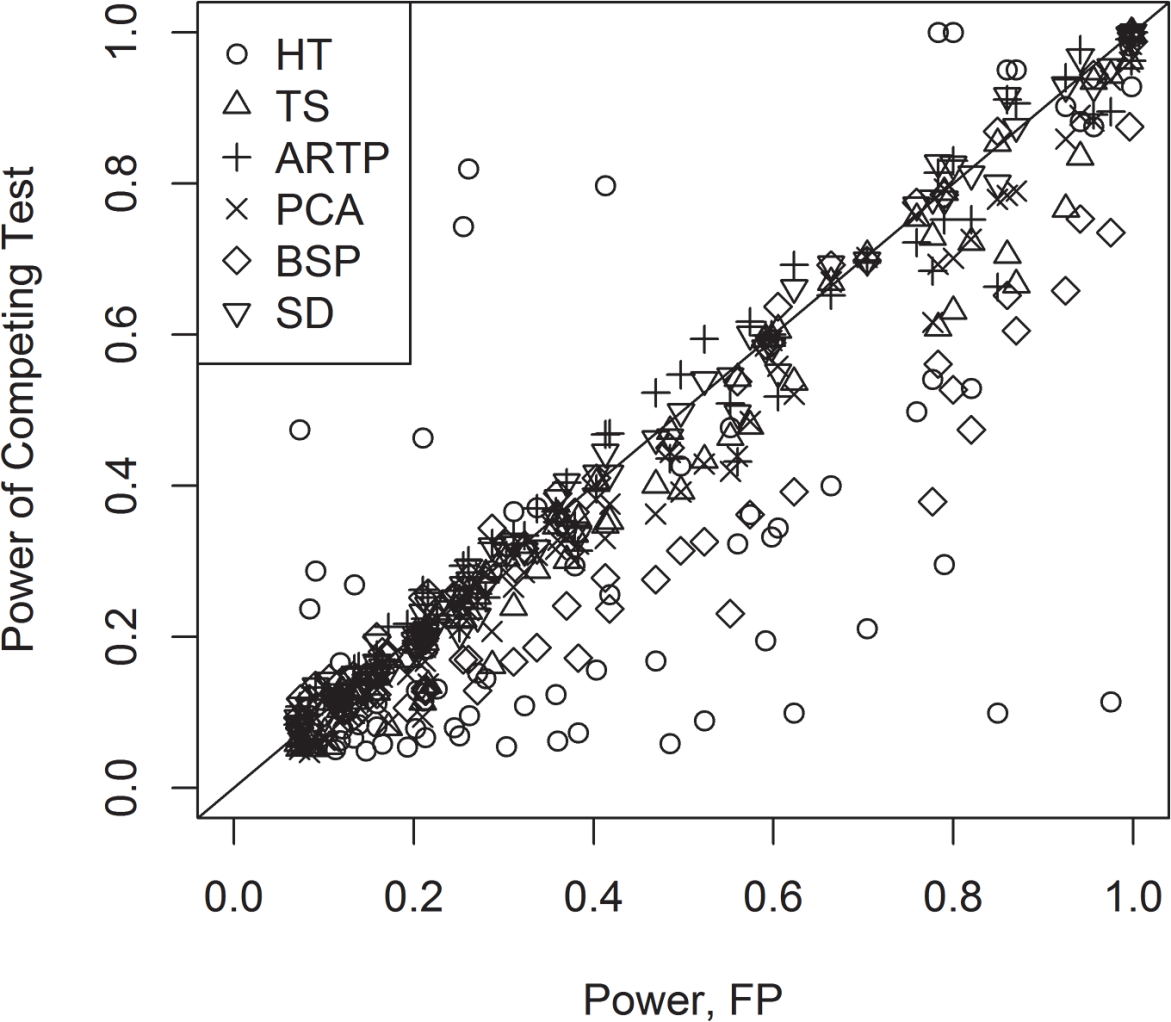
Comparison of the empirical power of Fisher’s statistic (FP) to the other statistics for the two-sided tests. The line y = x is shown for comparison to Fisher’s empirical power.

**Fig 3:**
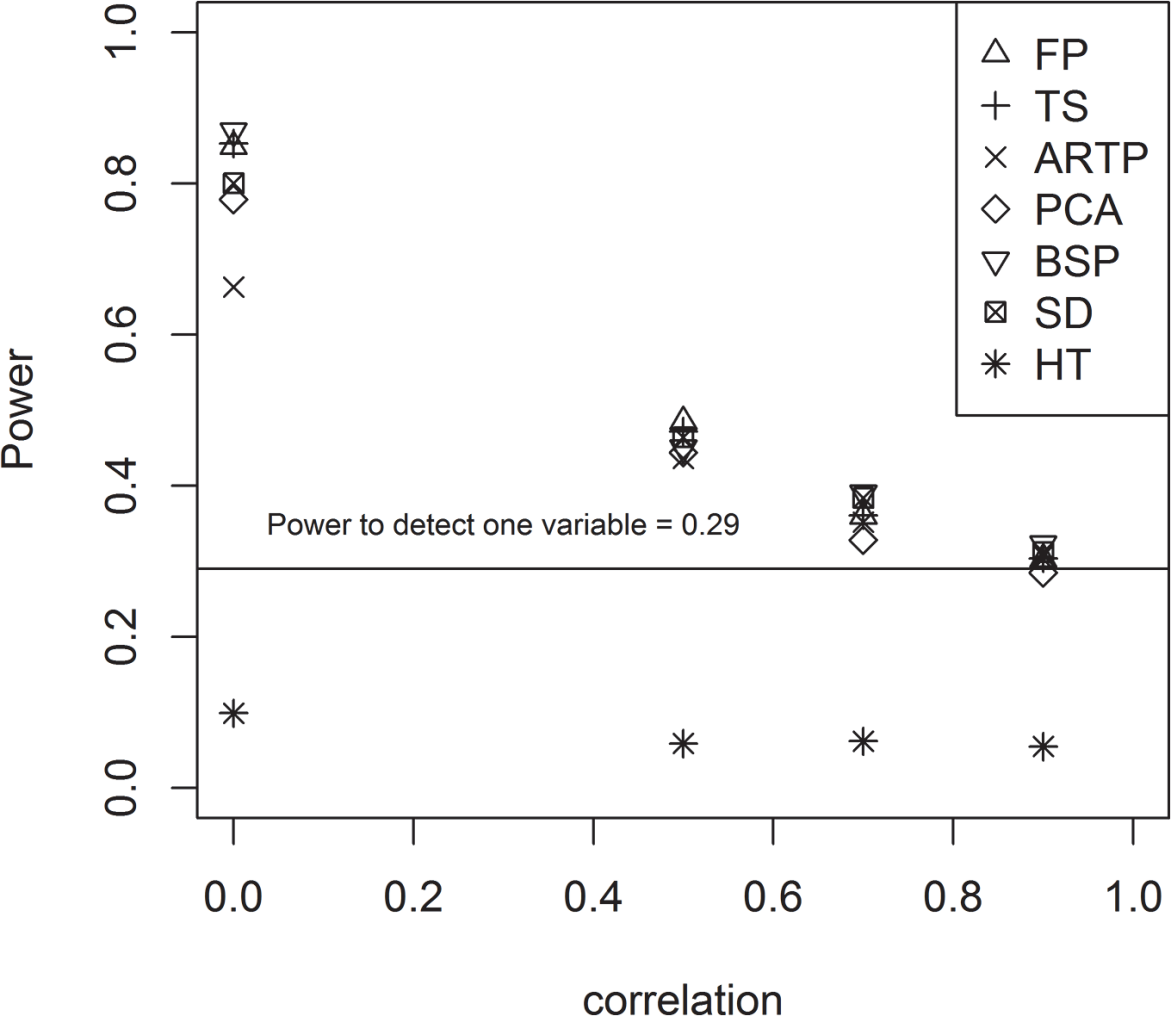
Comparison of the empirical power for the tests for 8 variables and two groups of 5 when all the mean changes are 0.3 and all of the standard deviations are 0.3. The vertical line shows the power to detect a single variable with a mean difference of 0.3 and standard deviation 0.3 (or the same ratio).

In the comparison of the p-value methods performed by Fridley et al., the Fisher statistic (FP) was the top overall performer [3]. Fig 2, shows the performance of the other tests relative to FP for the two-sided tests. Those above the line y = x have higher power than FP, and those below the line have lower power. From Fig 2, it is clear that Hotelling’s test (HT) performs much differently from the others. In many of the parameter combinations, it has much lower power than the other methods. To see this more specifically, Fig 3 shows the comparison of the empirical power for two groups of 5 subjects and 8 variables when all 8 elements of the mean vector are 0.3 (**m**_22_) and the standard deviations are 0.3 (**σ**_21_). The power to detect the change for a single variable is 0.29, and HT even performs worse than that (also see S1, S2, and S3 Tables). This low power is the result of the low sample size relative to the number of variables, as 10 subjects (two groups of 5) is the minimum number of subjects required for the inversion of the sample covariance matrix, and this is the only method that uses the inverse of the sample covariance matrix in the computation of the statistic.

There are some cases where the power for HT is much larger than the others. For example, when there are two groups of 10 subjects each, the power of HT to detect the mean difference of **m**_13_ = (0.3,0,0,0) with all standard deviations 0.3 (**σ**_11_) is much higher than the others, and much higher than its ability to detect **m**_12_ = (0.3,0.3,0.3,0.3)! Furthermore, this power increases with increasing correlation, when a decrease would be expected (see the bottom four rows of S1, S2, S4, and S5 Tables). This is very counterintuitive, but by examining the two-variable case, we can gain some insight. Suppose there are two variables with correlation ρ and standard deviation = 1. For the same sample sizes, we evaluate (substitute the sample means and sample covariance matrix in the test statistic with the expected values) ${\left(\mu_{11}-\mu_{12},\mu_{21}-\mu_{22}\right)}^{'}\sum^{-1}\left(\mu_{11}-\mu_{12},\mu_{21}-\mu_{22}\right)=\frac{1}{1-\rho^{2}}\left({\left(\mu_{11}-\mu_{12}\right)}^{2}+{\left(\mu_{21}-\mu_{22}\right)}^{2}-2\rho \left(\mu_{11}-\mu_{12}\right)\left(\mu_{21}-\mu_{22}\right)\right)$. Thus, when the mean difference for each variable is μ, this reduces to $\frac{2\mu^{2}\;\left(1-\rho \right)}{\left(1-\rho^{2}\right)}$ and if the mean difference for variable one is μ, but variable two is 0, then we have $\frac{\mu^{2}}{\left(1-\rho^{2}\right)}$. For independent variables this is 2μ^2^ vs. μ^2^. However, when ρ = 0.9, the first equation is approximately 1.05*μ^2^, while the second equation is approximately 5.26*μ^2^! This is definitely not a desirable property for the power. The other multivariate statistics behave as desired.

**Fig 4:**
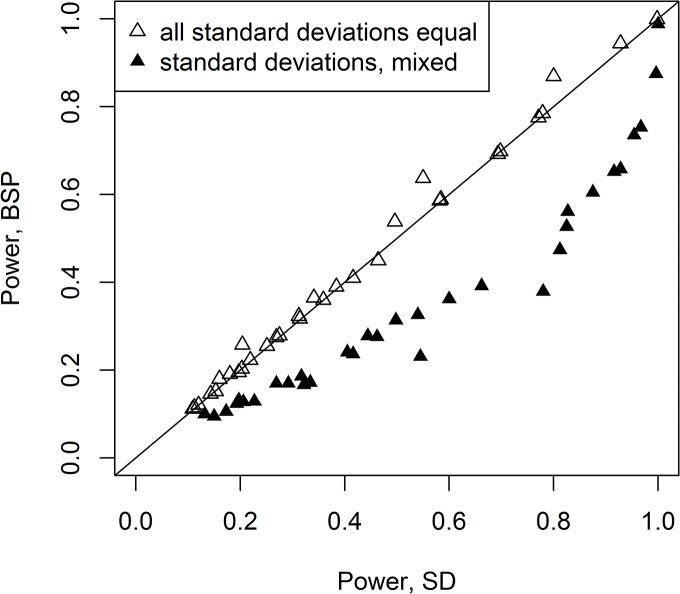
Comparison of the empirical power of Srivastava-Du ‘s test (SD) to Bai-Saranadasa’s test (BSP) for two-sided tests. This includes all the data from S1 and S2 Tables for these except for the case of one strong mean change (**m**_13_ and **m**_23_).

**Fig 5:**
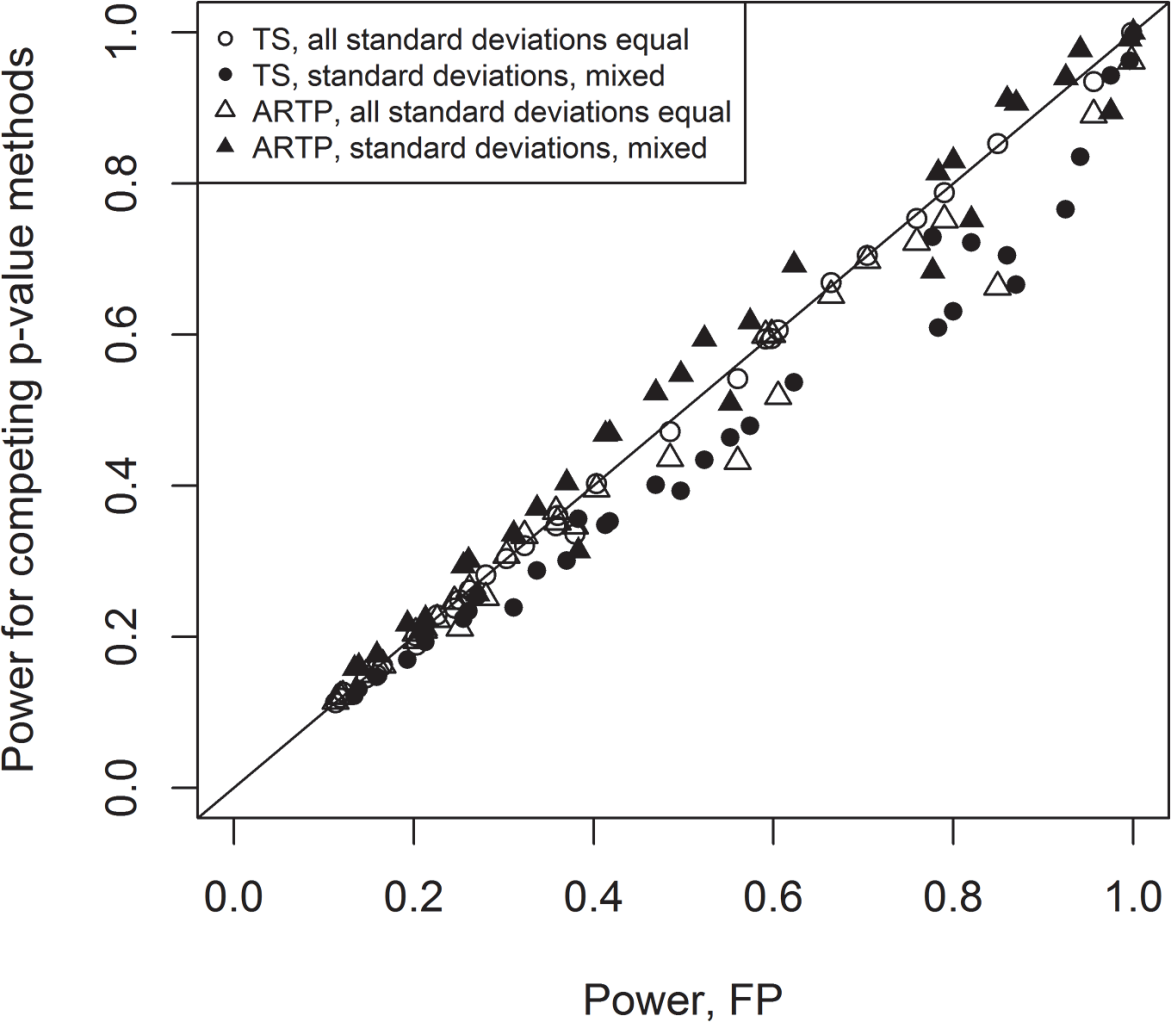
Comparison of the three p-value methods for the two-sided tests. This includes all the data from S1 and S2 Tables for these except for the case of one strong mean change (**m**_13_ and **m**_23_).

**Fig 6:**
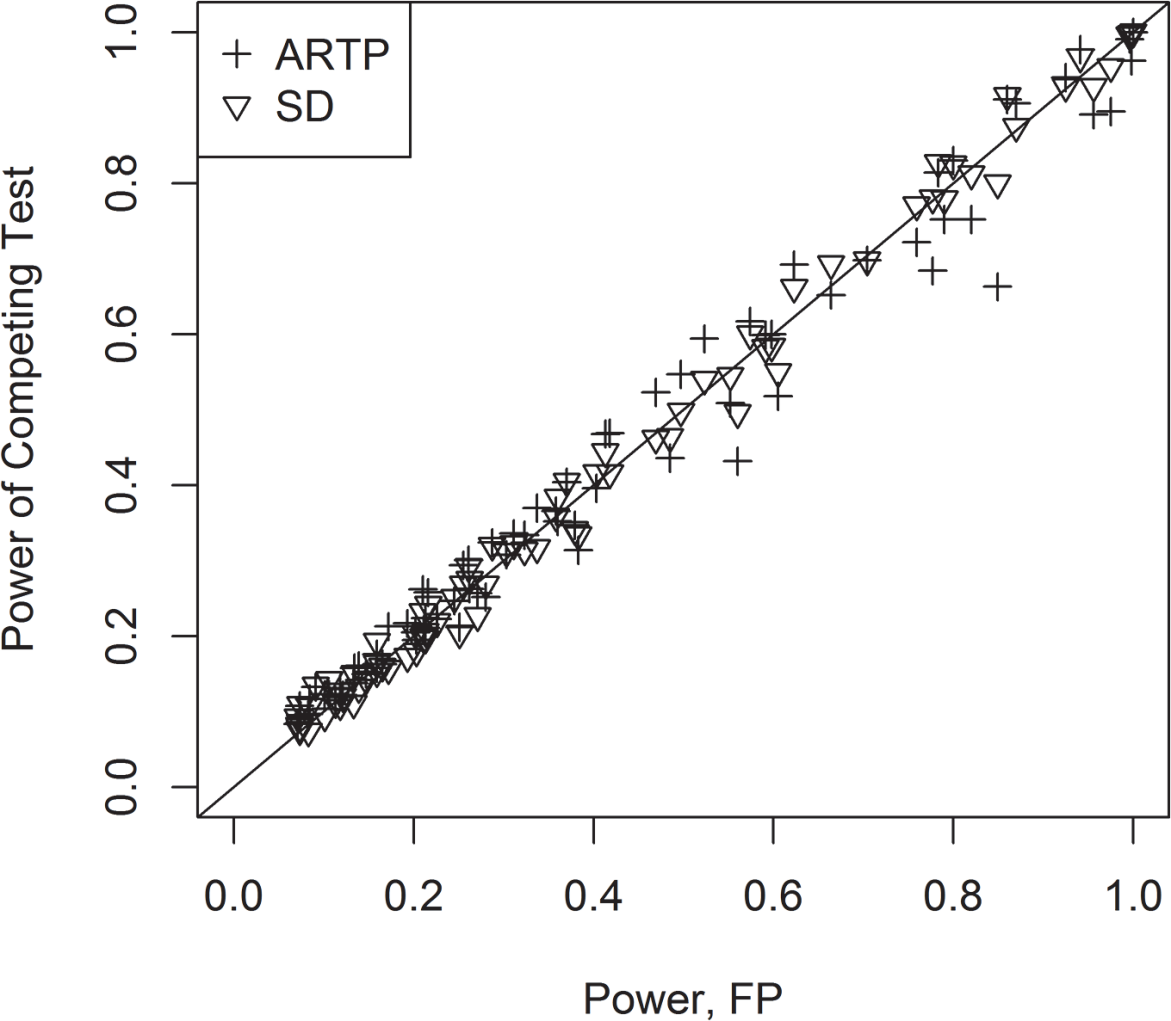
Comparison of the empirical power for the top 3 methods for two-sided tests.

From Fig 2, we see that SD and ARTP appear to be the most competitive methods to FP, while PCA, TS, BSP often underperform compared to the others. To see some of the patterns more clearly, the empirical power for SD compared to BSP is shown in Fig 4 for the two-sided tests for all combinations except for those where the means had only one non-zero element (**m**_13_ and **m**_23_). From Fig 4, one can clearly see that for the cases where the standard deviations for each variable are the same (**σ**_11_ and **σ**_21_), the two methods are comparable; however, when the standard deviations are mixed (**σ**_12_ and **σ**_22_), SD clearly outperforms BSP. In Fig 5, a similar comparison is made for the p-value methods with the line y = x indicating deviations from FP. For the mixed standard deviations, TS clearly underperforms FP and ARTP; but, for the mixed standard deviations, ARTP often outperforms FP, while for many cases where the standard deviations are the same, it underperforms FP. Fig 6 shows a comparison now with just the top 3 performers: FP, ARTP, and SD. Finally, to see the effect of larger samples sizes, FP was compared to SD for four variables with two groups of 20 subjects each and two groups with 50 subjects each: their empirical powers are virtually identical (see S6 Table).

**Fig 7:**
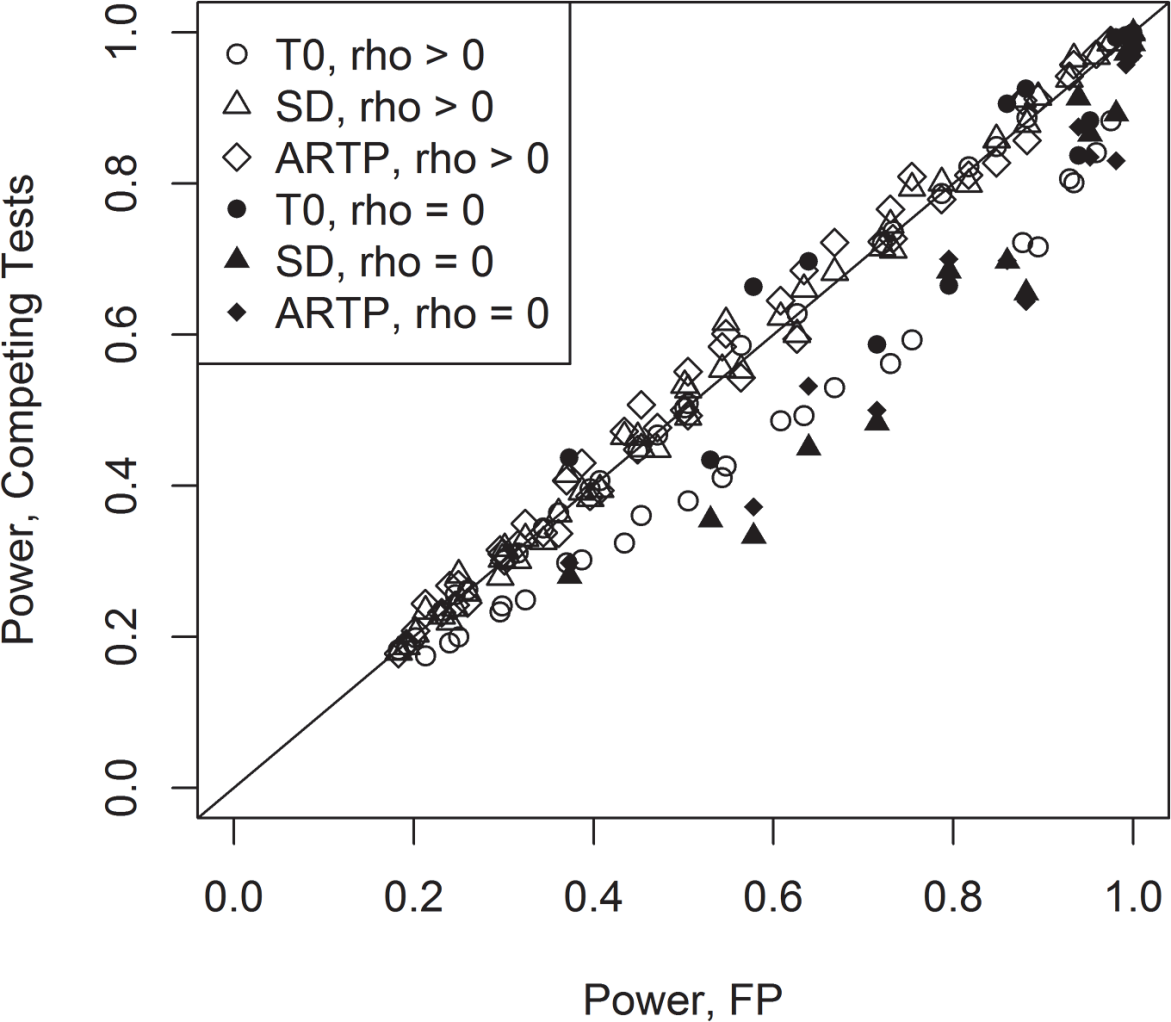
Comparison of the empirical power of Fisher’s test to the other top performers for one-sided alternatives. This includes all the data from S4 and S5 Tables for these except for the case of one strong mean change (**m**_13_ and **m**_23_).

For one-sided tests, comparing S1 Table to S4 Table and S2 Table to S5 Table, one can clearly see the improvement in power for the one-sided tests, as expected. Next, we compare the top 3 performers for the two-sided tests (FP, ARTP, SD) and T0. These are shown in Fig 7, with FP compared against the other three. Here, all combinations were used except for those where the mean vector contained only one non-zero element (**m**_13_ and **m**_23_). T0 generally underperforms against the others, except for independent variables. The others are fairly comparable, but FP consistently outperforms SD for the independent variables (but these two are similar for two-sided tests—see S1 and S2 Tables for the ρ = 0 rows).

Overall, we see that many of the methods are comparable for many of the combinations, but HT often has much lower power and sometimes lower than the power for a single variable. In most cases, the p-value methods perform at least as well as the multivariate statistics. In particular, FP is usually among the top ranking statistics. ARTP is competitive with FP when the variances are unequal. SD is typically the top performer for the multivariate statistics and is often comparable to the top p-value methods, with the exception of independent variables for the one-sided tests.

### Human Metabolomics Study

Table 3 shows a summary of the results from performing Welch’s two-sample t-test for each metabolite. As mentioned previously, any pathway where only one metabolite was observed was dropped from the analysis. There are 39 pathways remaining. Of these, only five pathways contain at least 10 metabolites, with 24 as the maximum. There is only one pathway, where every member is significant at the 0.05 level (P02 = benzoate metabolism, see Table 3 and S7 Table), and as expected, the p-values for assessing the significance of this pathway are very strong (Table 4). Results for pathways such as P01, where none of its individual members are even close to significant, and pathways such as P17, which has many strong p-values, have pathway statistics one would expect. The results for some of the other pathways are not obvious from simply examining the individual p-values. In particular, there are some cases where none of the individual p-values reach the threshold for significance, but the pathway statistic is indeed significant. Two such pathways are P13 (p-values 0.1334, 0.0540) and P31 (p-values 0.1332, 0.0563) whose p-values from Fisher’s statistic are 0.0449 and 0.0475, respectively. The individual p-values for P13 and P31 are similar to P08 (0.1444, 0.0633), but the p-value for P08 from Fisher’s statistic is 0.0857. The difference between these results can be explained by the differences in the correlations, which are positive in general (see S1 Fig). For the P13 and P31 pathways, the pair-wise correlations (computed from the pooled covariance matrices) are -0.09 and -0.08, respectively, so for both of these pathways, the two members are essentially independent. However, for P08, the pair-wise correlation between its two members is 0.81, showing that these two variables are highly redundant, and hence combining the signals does not strengthen the overall results.

**Table 3:** Summary of individual p-values.

PATHWAY	m	sig^a^	p-values
P01	4	0	0.6721, 0.6190, 0.5413, 0.4519
**P02** ^c^	3	3	0.0386, 1.17E-05, 2.41E-06
P03	2	0	0.4179, 0.2534
**P04**	2	1	0.0713, 2.95E-06
**P05**	2	1	0.3630, 0.0002
**P06**	8	4	0.6591, 0.6140, 0.4535, 0.2111,0.0407, 0.0366, 0.0002, 3.81E-05
P07	2	0	0.7851, 0.5707
P08	2	0	0.1444, 0.0633
**P09**	6	1	0.9781, 0.6044, 0.5859, 0.1883, 0.1103, 0.0032
**P10**	3	1	0.8279, 0.4513, 4.25E-07
**P11**	7	3	0.3994, 0.2128, 0.1814, 0.0534, 0.0468, 0.0354, 0.0272
P12	3	0	0.7530, 0.2264, 0.1326
**P13**	2	0	0.1334, 0.0540
**P14**	5	4	0.9990, 0.0358, 0.0145, 0.0043, 1.60E-07
**P15**	5	2	0.4057, 0.3830, 0.0572, 0.0085, 9.70E-05
P16	5	1	0.4169, 0.2648, 0.1696, 0.1653, 0.0008
**P17**	13	8	0.6672–0.1693 (5)^b^, 0.0429, 0.0082, 0.0006, 0.0019, 0.0006–3.22E-05 (4)
**P18**	11	4	0.7849–0.2877 (5), 0.1214, 0.0502, 0.0040, 0.0006, 0.0004, 6.70E-06
P19	4	0	0.8485, 0.4272, 0.3245, 0.2271
**P20**	24	14	0.7215–0.2160 (4), 0.1445–0.0618 (6), 0.0427–0.001 (9), < 0.0001 (5)
**P21**	7	2	0.9093, 0.4074, 0.1114, 0.0844, 0.0601, 0.0051, 0.0099
**P22**	5	3	0.9603, 0.3958, 0.0005, 2.20E-05, 1.73E-19
P23	2	1	0.2578, 0.0323
**P24**	2	1	0.5845, 1.50E-06
**P25**	8	3	0.9331, 0.6834, 0.6588, 0.3732, 0.1910, 0.0139, 0.0038, 1.24E-05
**P26**	2	1	0.0019, 0.3110
**P27**	3	2	0.3674, 0.0375, 0.003
**P28**	10	5	0.8412–0.5434, 0.3493, 0.1509, 0.0345, 0.0223, 0.0137, 0.0035, 5.15E-06
P29	3	0	0.7889, 0.5844, 0.5531
**P30**	3	1	0.4557, 0.0629, 2.15E-06
**P31**	2	0	0.1332, 0.0563
P32	2	0	0.7619, 0.2288
P33	6	0	0.9291, 0.7553, 0.5283, 0.2887, 0.2235, 0.0614
**P34**	14	5	0.7938–0.5069 (6), 0.1521–0.1046, 0.0361, 0.0207, 0.0196, 0.0065, 0.0042
P35	3	0	0.8001, 0.4088, 0.1320
**P36**	4	3	0.1851, 0.0291, 0.0204, 0.0201
**P37**	8	1	0.9430, 0.5743, 0.5072, 0.3653, 0.2682, 0.0911, 0.0629, 5.74E-05
P38	9	2	0.8732, 0.7545, 0.5775, 0.3838, 0.2843, 0.2286, 0.1660,0.0408, 0.0082
P39	4	1	0.8879, 0.4693, 0.3702, 0.0140

^a^ refers to the number of p-values < 0.05

^b^ (k) in the p-value column indicates that there are (k) p-values in the range

^c^ in bold are pathways significant with Fisher’s statistic

**Table 4:** p-values of the pathway statistics for the human metabolomics study.

CODE	m	FP	TS	ARTP	PCA	HT	BSP	DM	SD
P01	4	0.7924	0.8284	0.8734	0.6562	0.8335	0.7825	0.7825	0.8547
P02	3	<0.0001	5.00E-04	<0.0001	7.05E-07	4.21E-06	<0.0001	<0.0001	<0.0001
P03	2	0.3364	0.2839	0.3821	0.2270	0.4663	0.4228	0.4228	0.3682
P04	2	1.00E-04	0.0026	<0.0001	4.72E-05	1.34E-05	<0.0001	<0.0001	1.00E-04
P05	2	0.0021	0.0648	0.0011	0.0058	0.0006	3.00E-04	3.00E-04	0.001
P06	8	0.0048	0.0495	0.0018	0.0850	9.83E-11	0.0023	0.0023	0.0044
P07	2	0.8006	0.8016	0.7885	0.5578	0.8249	0.8563	0.8563	0.8167
P08	2	0.0857	0.0776	0.0855	0.0782	0.1434	0.074	0.074	0.0743
P09	6	0.0459	0.1229	0.0209	0.2546	0.0364	0.0098	0.0098	0.0279
P10	3	<0.0001	0.2276	<0.0001	0.0007	1.34E-05	0.0386	0.0374	<0.0001
P11	7	0.0411	0.0282	0.0741	0.0359	0.2915	0.0583	0.0583	0.046
P12	3	0.2697	0.2383	0.2667	0.3460	0.2085	0.1939	0.1938	0.3025
P13	2	0.0449	0.0211	0.0828	0.0190	0.0496	0.0301	0.0301	0.0403
P14	5	<0.0001	0.0065	<0.0001	3.70E-05	2.90E-08	<0.0001	<0.0001	<0.0001
P15	5	1.00E-04	0.0022	<0.0001	0.0001	0.0005	0.0282	0.0281	3.00E-04
P16	5	0.0504	0.049	0.0363	0.6688	0.0003	0.0138	0.0137	0.0532
P17	13	<0.0001	2.00E-04	1.00E-04	0.0009	9.69E-11	<0.0001	<0.0001	<0.0001
P18	11	0.0048	0.0595	0.0018	0.0090	1.41E-06	0.0114	0.0113	0.0046
P19	4	0.5114	0.4696	0.5776	0.7579	0.4641	0.3254	0.3254	0.54
P20	24	1.00E-04	5.00E-04	1.00E-04	0.0010	1.53E-14	1.00E-04	1.00E-04	1.00E-04
P21	7	0.0199	0.0326	0.0167	0.0205	0.0152	0.0434	0.0435	0.0282
P22	5	<0.0001	0.0138	<0.0001	5.46E-10	0	<0.0001	<0.0001	<0.0001
P23	2	0.0511	0.0409	0.0428	0.0398	0.0852	0.106	0.106	0.0577
P24	2	<0.0001	0.11	<0.0001	0.0040	7.06E-06	<0.0001	<0.0001	<0.0001
P25	8	3.00E-04	0.0468	<0.0001	0.7287	0.0002	0.0022	0.0021	1.00E-04
P26	2	0.0062	0.0294	0.0035	0.0064	0.0062	0.002	0.002	0.0062
P27	3	0.0039	0.0295	0.0016	0.6465	3.22E-14	0.0133	0.0129	0.005
P28	10	0.0064	0.0506	0.0026	0.0110	0.0001	0.0092	0.0091	0.006
P29	3	0.8175	0.8381	0.8701	0.7428	0.7852	0.8295	0.8295	0.8555
P30	3	<0.0001	0.0118	<0.0001	0.0015	6.94E-06	0.0301	0.0298	<0.0001
P31	2	0.0475	0.0222	0.0861	0.0222	0.0704	0.1178	0.1178	0.0618
P32	2	0.4858	0.4783	0.4397	0.3329	0.4987	0.3606	0.3606	0.4864
P33	6	0.3595	0.3355	0.2710	0.3099	0.3664	0.3533	0.3533	0.361
P34	14	0.0344	0.0613	0.0202	0.3507	2.29E-05	0.017	0.0167	0.0287
P35	3	0.3927	0.3534	0.3282	0.1892	0.3932	0.1291	0.1291	0.3595
P36	4	0.0019	<0.0001	0.0420	0.0048	0.0083	0.0222	0.0219	0.0023
P37	8	0.0078	0.0641	0.0024	0.0315	0.0011	0.014	0.0137	0.0044
P38	9	0.0604	0.0641	0.0321	0.0465	0.1798	0.1107	0.111	0.0581
P39	4	0.1835	0.2813	0.1436	0.4823	4.51E-05	0.0911	0.0904	0.1482

Although in most cases, the p-values are similar for the different methods, there are a few exceptions. For example, for pathway P39 none of the statistics for the pathway are significant at the 0.05-level (e.g., Fisher’s p-value is 0.1835), except for Hotelling’s statistic, which has a p-value < 0.0001. Of the four metabolites in this pathway, there is only one strong p-value, and the pair-wise correlations are 0.48, 0.51, 0.57, 0.84, 0.85, and 0.88. This seems to mirror the results from the simulation study where Hotelling’s statistic had stronger empirical powers than the others for the case of one strong change and highly correlated variables.

Overall, using the pathway analyses discussed, more than half the pathways are significant at the 0.05-level (before correcting for multiple comparisons) as seen in Table 4, with several pathways that are statistically significant where fewer than half the members reached the 0.05 level. As the sample sizes are very high here, it is not surprising that many of the measures give very similar results with the exception of PCA, which in some cases performed much worse. The p-values for the top performers from the simulation for two-sided tests, Fisher’s statistic (FP), the adaptive truncated product (ARTP), and Srivastava-Du (SD) have very similar p-values.

## Discussion

An extensive simulation study was performed to compare various statistics for assessing the statistical significance of a pathway. The statistics assessed were mainly either based on an aggregation of the p-values or multivariate statistics. While all the multivariate statistics require that the covariance matrix is the same for each group, most of the multivariate statistics considered do not require the inversion of the sample covariance matrix, and hence could be applied for even small sample sizes. The sample sizes and variables sizes in the simulation study were chosen to represent sizes seen in animal metabolomic studies. The empirical powers for the p-value aggregation methods (top performers were generally Fisher’s statistic and the ARTP statistic) are at least good as those from the multivariate statistics (top performer was the Srivastava-Du statistic). Furthermore, the power can be improved if appropriate one-sided statistics are implemented. With these sample sizes, permutation distributions are recommended over asymptotic approximations. Additionally, permutation statistics are recommended over test statistics that assume independent variables, such as the chi-squared statistic for Fisher’s test, since the variables within a pathway are often highly correlated. The main alternatives considered were those for changes for all metabolites in the pathway, rather than an alternative such as one metabolite is differential, while the others are not. Hence for other alternatives, the multivariate statistics may have an advantage. The p-value methods also have the advantage of being easier to adapt to various statistical designs, i.e., experimental designs that are more complicated than two independent groups.

## Supporting Information

S1 Fig Distribution of the pair-wise correlations from the 39 pathways for the insulin resistance data set. (TIFF)

S1 File Metabolomics data set used for the analysis. (CSV)

S1 Table Empirical Power, 4 variables, two-sided tests. (DOCX)

S2 Table Empirical Power, 8 variables, two-sided tests. (DOCX)

S3 Table Power for single variables for the two-sample t-test with the pooled variance estimate. (DOCX)

S4 Table Empirical Power, 4 variables, one-sided tests. (DOCX)

S5 Table Empirical Power, 8 variables one-sided tests. (DOCX)

S6 Table Two-sided t-tests, 4 variables, large samples: Fisher vs. Srivastava-Du. (DOCX)

S7 Table Pathway code from human metabolomics study. (DOCX)
