# Supplementary figures and images for: A Comparison of Aggregate P-Value Methods and Multivariate Statistics for Self-Contained Tests of Metabolic Pathway Analysis
Source: PLoS One. 2015 Apr 30;10(4):e0125081. doi: 10.1371/journal.pone.0125081 (PMC4415974; doi:10.1371/journal.pone.0125081)

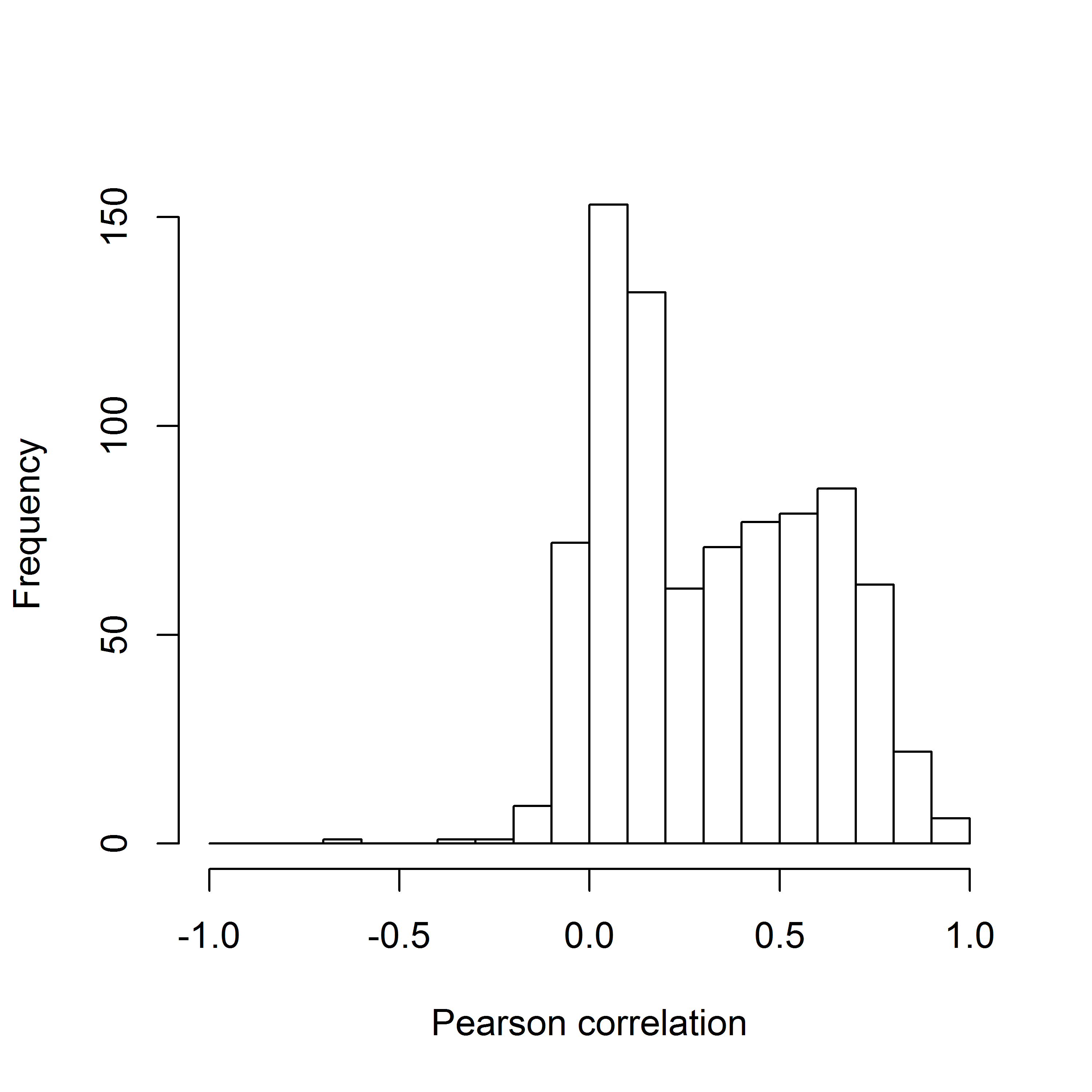

Supplement: S1 Fig — (TIFF) [file pone.0125081.s001.tiff]
