# Supplementary material for: A Comparison of Aggregate P-Value Methods and Multivariate Statistics for Self-Contained Tests of Metabolic Pathway Analysis
Source: PLoS One. 2015 Apr 30;10(4):e0125081. doi: 10.1371/journal.pone.0125081 (PMC4415974; doi:10.1371/journal.pone.0125081)
Supplement: S1 Table — (DOCX) [file pone.0125081.s003.docx]

S_Table 1: Empirical Power, 4 variables, two-sided tests

| MU | σ | ρ | N | FP | TS | ARTP | PCA | HT | BSP | DM | SD |
| --- | --- | --- | --- | --- | --- | --- | --- | --- | --- | --- | --- |
| m11 | σ11 | 0.9 | 5 | 0.118 | 0.119 | 0.121 | 0.103 | 0.063 | 0.112 | 0.11 | 0.109 |
| m11 | σ12 | 0.9 | 5 | 0.134 | 0.122 | 0.158 | 0.099 | 0.269 | 0.095 | 0.097 | 0.15 |
| m11 | σ11 | 0.7 | 5 | 0.121 | 0.127 | 0.122 | 0.114 | 0.078 | 0.121 | 0.118 | 0.12 |
| m11 | σ12 | 0.7 | 5 | 0.159 | 0.147 | 0.176 | 0.13 | 0.145 | 0.124 | 0.127 | 0.193 |
| m11 | σ11 | 0.5 | 5 | 0.159 | 0.15 | 0.163 | 0.138 | 0.081 | 0.152 | 0.15 | 0.153 |
| m11 | σ12 | 0.5 | 5 | 0.213 | 0.193 | 0.211 | 0.168 | 0.126 | 0.132 | 0.132 | 0.197 |
| m11 | σ11 | 0 | 5 | 0.203 | 0.189 | 0.195 | 0.169 | 0.129 | 0.191 | 0.185 | 0.18 |
| m11 | σ12 | 0 | 5 | 0.271 | 0.254 | 0.257 | 0.247 | 0.152 | 0.129 | 0.136 | 0.227 |
| m12 | σ11 | 0.9 | 5 | 0.323 | 0.321 | 0.334 | 0.285 | 0.109 | 0.317 | 0.317 | 0.314 |
| m12 | σ12 | 0.9 | 5 | 0.413 | 0.348 | 0.468 | 0.33 | 0.797 | 0.278 | 0.283 | 0.444 |
| m12 | σ11 | 0.7 | 5 | 0.358 | 0.347 | 0.366 | 0.316 | 0.124 | 0.36 | 0.358 | 0.359 |
| m12 | σ12 | 0.7 | 5 | 0.497 | 0.393 | 0.547 | 0.392 | 0.426 | 0.314 | 0.319 | 0.498 |
| m12 | σ11 | 0.5 | 5 | 0.403 | 0.403 | 0.396 | 0.382 | 0.156 | 0.41 | 0.409 | 0.416 |
| m12 | σ12 | 0.5 | 5 | 0.574 | 0.479 | 0.617 | 0.485 | 0.362 | 0.362 | 0.375 | 0.6 |
| m12 | σ11 | 0 | 5 | 0.605 | 0.606 | 0.518 | 0.558 | 0.344 | 0.637 | 0.623 | 0.55 |
| m12 | σ12 | 0 | 5 | 0.82 | 0.722 | 0.752 | 0.726 | 0.529 | 0.474 | 0.497 | 0.812 |
| m13 | σ11 | 0.9 | 5 | 0.101 | 0.073 | 0.123 | 0.063 | 0.602 | 0.1 | 0.108 | 0.094 |
| m13 | σ11 | 0.7 | 5 | 0.084 | 0.056 | 0.117 | 0.047 | 0.237 | 0.118 | 0.129 | 0.107 |
| m13 | σ11 | 0.5 | 5 | 0.118 | 0.104 | 0.131 | 0.087 | 0.166 | 0.139 | 0.15 | 0.127 |
| m13 | σ11 | 0 | 5 | 0.159 | 0.127 | 0.158 | 0.114 | 0.112 | 0.2 | 0.201 | 0.164 |
| m11 | σ11 | 0.9 | 10 | 0.211 | 0.206 | 0.209 | 0.207 | 0.115 | 0.195 | 0.196 | 0.197 |
| m11 | σ12 | 0.9 | 10 | 0.255 | 0.224 | 0.294 | 0.226 | 0.743 | 0.17 | 0.171 | 0.269 |
| m11 | σ11 | 0.7 | 10 | 0.226 | 0.229 | 0.223 | 0.224 | 0.131 | 0.223 | 0.221 | 0.219 |
| m11 | σ12 | 0.7 | 10 | 0.311 | 0.239 | 0.336 | 0.266 | 0.366 | 0.167 | 0.169 | 0.322 |
| m11 | σ11 | 0.5 | 10 | 0.28 | 0.282 | 0.252 | 0.285 | 0.145 | 0.275 | 0.277 | 0.269 |
| m11 | σ12 | 0.5 | 10 | 0.37 | 0.301 | 0.404 | 0.331 | 0.343 | 0.241 | 0.245 | 0.405 |
| m11 | σ11 | 0 | 10 | 0.379 | 0.336 | 0.347 | 0.301 | 0.294 | 0.365 | 0.363 | 0.341 |
| m11 | σ12 | 0 | 10 | 0.552 | 0.464 | 0.509 | 0.419 | 0.477 | 0.231 | 0.24 | 0.545 |
| m12 | σ11 | 0.9 | 10 | 0.598 | 0.595 | 0.6 | 0.592 | 0.332 | 0.589 | 0.587 | 0.584 |
| m12 | σ12 | 0.9 | 10 | 0.783 | 0.609 | 0.814 | 0.693 | 1 | 0.561 | 0.575 | 0.827 |
| m12 | σ11 | 0.7 | 10 | 0.664 | 0.669 | 0.652 | 0.67 | 0.4 | 0.692 | 0.693 | 0.693 |
| m12 | σ12 | 0.7 | 10 | 0.87 | 0.666 | 0.906 | 0.79 | 0.95 | 0.605 | 0.614 | 0.875 |
| m12 | σ11 | 0.5 | 10 | 0.759 | 0.754 | 0.722 | 0.757 | 0.498 | 0.775 | 0.769 | 0.771 |
| m12 | σ12 | 0.5 | 10 | 0.925 | 0.766 | 0.94 | 0.859 | 0.902 | 0.658 | 0.674 | 0.928 |
| m12 | σ11 | 0 | 10 | 0.956 | 0.935 | 0.891 | 0.883 | 0.875 | 0.944 | 0.943 | 0.928 |
| m12 | σ12 | 0 | 10 | 0.996 | 0.962 | 0.991 | 0.961 | 0.982 | 0.875 | 0.887 | 0.996 |
| m13 | σ11 | 0.9 | 10 | 0.106 | 0.055 | 0.123 | 0.062 | 0.992 | 0.145 | 0.158 | 0.142 |
| m13 | σ11 | 0.7 | 10 | 0.172 | 0.08 | 0.213 | 0.086 | 0.697 | 0.176 | 0.193 | 0.157 |
| m13 | σ11 | 0.5 | 10 | 0.21 | 0.114 | 0.262 | 0.094 | 0.463 | 0.252 | 0.272 | 0.232 |
| m13 | σ11 | 0 | 10 | 0.287 | 0.162 | 0.324 | 0.207 | 0.288 | 0.344 | 0.345 | 0.32 |
