# Supplementary material for: A Comparison of Aggregate P-Value Methods and Multivariate Statistics for Self-Contained Tests of Metabolic Pathway Analysis
Source: PLoS One. 2015 Apr 30;10(4):e0125081. doi: 10.1371/journal.pone.0125081 (PMC4415974; doi:10.1371/journal.pone.0125081)
Supplement: S2 Table — (DOCX) [file pone.0125081.s004.docx]

S _Table 2: Empirical Power, 8 variables, two-sided tests

| MU | σ | ρ | n | FP | PCA | TS | ARTP | HT | BSP | DM | SD |
| --- | --- | --- | --- | --- | --- | --- | --- | --- | --- | --- | --- |
| m21 | σ21 | 0.9 | 5 | 0.113 | 0.097 | 0.113 | 0.115 | 0.051 | 0.115 | 0.117 | 0.112 |
| m21 | σ22 | 0.9 | 5 | 0.139 | 0.117 | 0.131 | 0.161 | 0.084 | 0.1 | 0.101 | 0.132 |
| m21 | σ21 | 0.7 | 5 | 0.147 | 0.123 | 0.146 | 0.15 | 0.049 | 0.146 | 0.147 | 0.143 |
| m21 | σ22 | 0.7 | 5 | 0.193 | 0.15 | 0.17 | 0.217 | 0.054 | 0.106 | 0.108 | 0.173 |
| m21 | σ21 | 0.5 | 5 | 0.165 | 0.147 | 0.162 | 0.163 | 0.058 | 0.18 | 0.176 | 0.16 |
| m21 | σ22 | 0.5 | 5 | 0.213 | 0.186 | 0.208 | 0.224 | 0.067 | 0.128 | 0.131 | 0.206 |
| m21 | σ21 | 0 | 5 | 0.251 | 0.212 | 0.249 | 0.212 | 0.069 | 0.258 | 0.258 | 0.204 |
| m21 | σ22 | 0 | 5 | 0.383 | 0.323 | 0.356 | 0.314 | 0.073 | 0.172 | 0.184 | 0.334 |
| m22 | σ21 | 0.9 | 5 | 0.303 | 0.285 | 0.304 | 0.308 | 0.055 | 0.323 | 0.324 | 0.312 |
| m22 | σ22 | 0.9 | 5 | 0.469 | 0.363 | 0.401 | 0.523 | 0.168 | 0.276 | 0.281 | 0.462 |
| m22 | σ21 | 0.7 | 5 | 0.36 | 0.328 | 0.361 | 0.352 | 0.062 | 0.39 | 0.389 | 0.384 |
| m22 | σ22 | 0.7 | 5 | 0.523 | 0.429 | 0.434 | 0.594 | 0.089 | 0.326 | 0.344 | 0.54 |
| m22 | σ21 | 0.5 | 5 | 0.485 | 0.444 | 0.472 | 0.436 | 0.059 | 0.45 | 0.455 | 0.464 |
| m22 | σ22 | 0.5 | 5 | 0.623 | 0.521 | 0.537 | 0.692 | 0.099 | 0.392 | 0.416 | 0.662 |
| m22 | σ21 | 0 | 5 | 0.849 | 0.779 | 0.853 | 0.663 | 0.099 | 0.869 | 0.873 | 0.8 |
| m22 | σ22 | 0 | 5 | 0.975 | 0.937 | 0.943 | 0.895 | 0.114 | 0.735 | 0.761 | 0.954 |
| m23 | σ21 | 0.9 | 5 | 0.083 | 0.056 | 0.066 | 0.097 | 0.105 | 0.076 | 0.082 | 0.074 |
| m23 | σ21 | 0.7 | 5 | 0.073 | 0.057 | 0.065 | 0.092 | 0.079 | 0.08 | 0.09 | 0.08 |
| m23 | σ21 | 0.5 | 5 | 0.074 | 0.056 | 0.068 | 0.095 | 0.064 | 0.118 | 0.12 | 0.109 |
| m23 | σ21 | 0 | 5 | 0.133 | 0.099 | 0.115 | 0.138 | 0.066 | 0.149 | 0.146 | 0.111 |
| m21 | σ21 | 0.9 | 10 | 0.202 | 0.201 | 0.201 | 0.205 | 0.079 | 0.203 | 0.203 | 0.203 |
| m21 | σ22 | 0.9 | 10 | 0.261 | 0.235 | 0.234 | 0.301 | 0.819 | 0.17 | 0.171 | 0.292 |
| m21 | σ21 | 0.7 | 10 | 0.245 | 0.241 | 0.238 | 0.247 | 0.08 | 0.255 | 0.252 | 0.251 |
| m21 | σ22 | 0.7 | 10 | 0.337 | 0.308 | 0.288 | 0.37 | 0.371 | 0.186 | 0.186 | 0.317 |
| m21 | σ21 | 0.5 | 10 | 0.262 | 0.264 | 0.262 | 0.263 | 0.096 | 0.279 | 0.278 | 0.275 |
| m21 | σ22 | 0.5 | 10 | 0.418 | 0.376 | 0.353 | 0.469 | 0.256 | 0.237 | 0.239 | 0.416 |
| m21 | σ21 | 0 | 10 | 0.56 | 0.439 | 0.542 | 0.432 | 0.323 | 0.538 | 0.541 | 0.496 |
| m21 | σ22 | 0 | 10 | 0.777 | 0.616 | 0.729 | 0.684 | 0.541 | 0.379 | 0.397 | 0.78 |
| m22 | σ21 | 0.9 | 10 | 0.591 | 0.585 | 0.594 | 0.599 | 0.195 | 0.586 | 0.586 | 0.582 |
| m22 | σ22 | 0.9 | 10 | 0.8 | 0.701 | 0.631 | 0.83 | 1 | 0.527 | 0.537 | 0.825 |
| m22 | σ21 | 0.7 | 10 | 0.704 | 0.702 | 0.705 | 0.698 | 0.211 | 0.698 | 0.701 | 0.698 |
| m22 | σ22 | 0.7 | 10 | 0.86 | 0.784 | 0.705 | 0.911 | 0.95 | 0.652 | 0.663 | 0.916 |
| m22 | σ21 | 0.5 | 10 | 0.79 | 0.793 | 0.788 | 0.752 | 0.296 | 0.785 | 0.785 | 0.779 |
| m22 | σ22 | 0.5 | 10 | 0.941 | 0.89 | 0.835 | 0.977 | 0.882 | 0.753 | 0.761 | 0.967 |
| m22 | σ21 | 0 | 10 | 0.998 | 0.984 | 1 | 0.962 | 0.928 | 0.999 | 0.999 | 0.998 |
| m22 | σ22 | 0 | 10 | 1 | 0.997 | 0.997 | 1 | 0.994 | 0.988 | 0.991 | 1 |
| m23 | σ21 | 0.9 | 10 | 0.071 | 0.06 | 0.059 | 0.084 | 0.954 | 0.093 | 0.098 | 0.093 |
| m23 | σ21 | 0.7 | 10 | 0.073 | 0.051 | 0.053 | 0.108 | 0.474 | 0.079 | 0.087 | 0.076 |
| m23 | σ21 | 0.5 | 10 | 0.091 | 0.059 | 0.052 | 0.133 | 0.287 | 0.136 | 0.144 | 0.135 |
| m23 | σ21 | 0 | 10 | 0.216 | 0.138 | 0.136 | 0.258 | 0.184 | 0.258 | 0.257 | 0.242 |
