# Supplementary material for: A Comparison of Aggregate P-Value Methods and Multivariate Statistics for Self-Contained Tests of Metabolic Pathway Analysis
Source: PLoS One. 2015 Apr 30;10(4):e0125081. doi: 10.1371/journal.pone.0125081 (PMC4415974; doi:10.1371/journal.pone.0125081)
Supplement: S3 Table — (DOCX) [file pone.0125081.s005.docx]

S_Table 3: Power for single variables for the two-sample t-test with the pooled variance estimate

| µ1-µ2 | σ | power, two-sided | power, one-sided |
| --- | --- | --- | --- |
| 0.15 | 0.15 | 0.29 | 0.42 |
| 0.15 | 0.25 | 0.13 | 0.22 |
| 0.15 | 0.3 | 0.10 | 0.18 |
| 0.15 | 0.35 | 0.09 | 0.15 |
| 0.15 | 0.45 | 0.07 | 0.12 |
| 0.3 | 0.15 | 0.79 | 0.89 |
| 0.3 | 0.25 | 0.39 | 0.54 |
| 0.3 | 0.3 | 0.29 | 0.42 |
| 0.3 | 0.35 | 0.22 | 0.34 |
| 0.3 | 0.45 | 0.15 | 0.25 |
| 0.15 | 0.15 | 0.56 | 0.69 |
| 0.15 | 0.25 | 0.25 | 0.36 |
| 0.15 | 0.3 | 0.18 | 0.28 |
| 0.15 | 0.35 | 0.15 | 0.24 |
| 0.15 | 0.45 | 0.10 | 0.18 |
| 0.3 | 0.15 | 0.99 | 1.00 |
| 0.3 | 0.25 | 0.72 | 0.83 |
| 0.3 | 0.3 | 0.56 | 0.69 |
| 0.3 | 0.35 | 0.44 | 0.58 |
| 0.3 | 0.45 | 0.29 | 0.42 |
