# Supplementary material for: A Comparison of Aggregate P-Value Methods and Multivariate Statistics for Self-Contained Tests of Metabolic Pathway Analysis
Source: PLoS One. 2015 Apr 30;10(4):e0125081. doi: 10.1371/journal.pone.0125081 (PMC4415974; doi:10.1371/journal.pone.0125081)
Supplement: S4 Table — (DOCX) [file pone.0125081.s006.docx]

S_Table 4: Empirical Power, 4 variables, one-sided tests

| MU | σ | ρ | N | FP | TS | ARTP | HT | BSP | DM | SD | T0 |
| --- | --- | --- | --- | --- | --- | --- | --- | --- | --- | --- | --- |
| m11 | σ11 | 0.9 | 5 | 0.202 | 0.2 | 0.208 | 0.105 | 0.201 | 0.202 | 0.204 | 0.199 |
| m11 | σ12 | 0.9 | 5 | 0.213 | 0.208 | 0.244 | 0.347 | 0.157 | 0.157 | 0.234 | 0.175 |
| m11 | σ11 | 0.7 | 5 | 0.192 | 0.191 | 0.191 | 0.131 | 0.186 | 0.184 | 0.187 | 0.189 |
| m11 | σ12 | 0.7 | 5 | 0.25 | 0.235 | 0.269 | 0.223 | 0.188 | 0.192 | 0.282 | 0.2 |
| m11 | σ11 | 0.5 | 5 | 0.246 | 0.25 | 0.242 | 0.137 | 0.238 | 0.237 | 0.238 | 0.256 |
| m11 | σ12 | 0.5 | 5 | 0.298 | 0.28 | 0.31 | 0.195 | 0.191 | 0.193 | 0.303 | 0.241 |
| m11 | σ11 | 0 | 5 | 0.373 | 0.425 | 0.298 | 0.22 | 0.304 | 0.314 | 0.28 | 0.437 |
| m11 | σ12 | 0 | 5 | 0.53 | 0.545 | 0.434 | 0.248 | 0.202 | 0.209 | 0.355 | 0.434 |
| m12 | σ11 | 0.9 | 5 | 0.471 | 0.47 | 0.477 | 0.204 | 0.452 | 0.451 | 0.448 | 0.467 |
| m12 | σ12 | 0.9 | 5 | 0.547 | 0.486 | 0.601 | 0.885 | 0.401 | 0.41 | 0.616 | 0.426 |
| m12 | σ11 | 0.7 | 5 | 0.505 | 0.502 | 0.493 | 0.235 | 0.502 | 0.498 | 0.491 | 0.509 |
| m12 | σ12 | 0.7 | 5 | 0.634 | 0.564 | 0.685 | 0.601 | 0.457 | 0.467 | 0.66 | 0.493 |
| m12 | σ11 | 0.5 | 5 | 0.564 | 0.59 | 0.543 | 0.273 | 0.563 | 0.561 | 0.553 | 0.586 |
| m12 | σ12 | 0.5 | 5 | 0.73 | 0.637 | 0.766 | 0.554 | 0.502 | 0.512 | 0.745 | 0.562 |
| m12 | σ11 | 0 | 5 | 0.86 | 0.885 | 0.698 | 0.497 | 0.757 | 0.753 | 0.697 | 0.905 |
| m12 | σ12 | 0 | 5 | 0.939 | 0.918 | 0.875 | 0.74 | 0.662 | 0.684 | 0.913 | 0.837 |
| m13 | σ11 | 0.9 | 5 | 0.134 | 0.11 | 0.162 | 0.505 | 0.139 | 0.15 | 0.126 | 0.13 |
| m13 | σ11 | 0.7 | 5 | 0.117 | 0.08 | 0.148 | 0.262 | 0.17 | 0.175 | 0.152 | 0.103 |
| m13 | σ11 | 0.5 | 5 | 0.146 | 0.122 | 0.179 | 0.205 | 0.193 | 0.204 | 0.182 | 0.137 |
| m13 | σ11 | 0 | 5 | 0.216 | 0.172 | 0.238 | 0.185 | 0.254 | 0.255 | 0.223 | 0.192 |
| m11 | σ11 | 0.9 | 10 | 0.316 | 0.315 | 0.322 | 0.178 | 0.303 | 0.303 | 0.301 | 0.311 |
| m11 | σ12 | 0.9 | 10 | 0.387 | 0.349 | 0.43 | 0.759 | 0.263 | 0.267 | 0.392 | 0.302 |
| m11 | σ11 | 0.7 | 10 | 0.344 | 0.339 | 0.338 | 0.207 | 0.325 | 0.324 | 0.326 | 0.344 |
| m11 | σ12 | 0.7 | 10 | 0.434 | 0.377 | 0.472 | 0.475 | 0.284 | 0.286 | 0.465 | 0.324 |
| m11 | σ11 | 0.5 | 10 | 0.396 | 0.4 | 0.386 | 0.245 | 0.38 | 0.38 | 0.383 | 0.396 |
| m11 | σ12 | 0.5 | 10 | 0.505 | 0.438 | 0.551 | 0.456 | 0.348 | 0.351 | 0.527 | 0.38 |
| m11 | σ11 | 0 | 10 | 0.639 | 0.678 | 0.532 | 0.424 | 0.489 | 0.486 | 0.45 | 0.697 |
| m11 | σ12 | 0 | 10 | 0.795 | 0.791 | 0.7 | 0.616 | 0.376 | 0.384 | 0.684 | 0.665 |
| m12 | σ11 | 0.9 | 10 | 0.733 | 0.733 | 0.727 | 0.473 | 0.711 | 0.71 | 0.712 | 0.737 |
| m12 | σ12 | 0.9 | 10 | 0.877 | 0.745 | 0.91 | 0.989 | 0.684 | 0.688 | 0.911 | 0.722 |
| m12 | σ11 | 0.7 | 10 | 0.787 | 0.784 | 0.779 | 0.548 | 0.8 | 0.799 | 0.801 | 0.787 |
| m12 | σ12 | 0.7 | 10 | 0.929 | 0.823 | 0.942 | 0.976 | 0.75 | 0.755 | 0.938 | 0.806 |
| m12 | σ11 | 0.5 | 10 | 0.848 | 0.838 | 0.827 | 0.64 | 0.863 | 0.86 | 0.858 | 0.849 |
| m12 | σ12 | 0.5 | 10 | 0.959 | 0.868 | 0.971 | 0.95 | 0.789 | 0.794 | 0.968 | 0.841 |
| m12 | σ11 | 0 | 10 | 0.992 | 0.993 | 0.957 | 0.942 | 0.976 | 0.975 | 0.972 | 0.996 |
| m12 | σ12 | 0 | 10 | 1 | 0.994 | 1 | 0.992 | 0.947 | 0.952 | 0.998 | 0.991 |
| m13 | σ11 | 0.9 | 10 | 0.161 | 0.078 | 0.198 | 0.718 | 0.226 | 0.243 | 0.225 | 0.12 |
| m13 | σ11 | 0.7 | 10 | 0.23 | 0.119 | 0.277 | 0.614 | 0.279 | 0.299 | 0.263 | 0.171 |
| m13 | σ11 | 0.5 | 10 | 0.212 | 0.107 | 0.273 | 0.46 | 0.327 | 0.335 | 0.311 | 0.156 |
| m13 | σ11 | 0 | 10 | 0.395 | 0.206 | 0.442 | 0.405 | 0.437 | 0.441 | 0.435 | 0.281 |
