# Supplementary material for: A Comparison of Aggregate P-Value Methods and Multivariate Statistics for Self-Contained Tests of Metabolic Pathway Analysis
Source: PLoS One. 2015 Apr 30;10(4):e0125081. doi: 10.1371/journal.pone.0125081 (PMC4415974; doi:10.1371/journal.pone.0125081)
Supplement: S5 Table — (DOCX) [file pone.0125081.s007.docx]

S_Table 5: Empirical Power, one-sided tests, 8 variables

| MU | σ | ρ | n | FP | TS | ARTP | HT | BSP | DM | SD | T0 |
| --- | --- | --- | --- | --- | --- | --- | --- | --- | --- | --- | --- |
| m21 | σ21 | 0.9 | 5 | 0.183 | 0.179 | 0.178 | 0.092 | 0.18 | 0.18 | 0.18 | 0.183 |
| m21 | σ22 | 0.9 | 5 | 0.24 | 0.232 | 0.268 | 0.141 | 0.159 | 0.16 | 0.22 | 0.192 |
| m21 | σ21 | 0.7 | 5 | 0.231 | 0.236 | 0.232 | 0.099 | 0.224 | 0.223 | 0.228 | 0.234 |
| m21 | σ22 | 0.7 | 5 | 0.296 | 0.272 | 0.315 | 0.093 | 0.175 | 0.177 | 0.279 | 0.233 |
| m21 | σ21 | 0.5 | 5 | 0.26 | 0.258 | 0.245 | 0.093 | 0.267 | 0.271 | 0.258 | 0.262 |
| m21 | σ22 | 0.5 | 5 | 0.324 | 0.311 | 0.35 | 0.1 | 0.2 | 0.202 | 0.33 | 0.249 |
| m21 | σ21 | 0 | 5 | 0.578 | 0.644 | 0.372 | 0.135 | 0.373 | 0.38 | 0.333 | 0.663 |
| m21 | σ22 | 0 | 5 | 0.715 | 0.749 | 0.5 | 0.147 | 0.272 | 0.28 | 0.483 | 0.587 |
| m22 | σ21 | 0.9 | 5 | 0.449 | 0.452 | 0.448 | 0.114 | 0.466 | 0.467 | 0.464 | 0.446 |
| m22 | σ22 | 0.9 | 5 | 0.608 | 0.563 | 0.645 | 0.281 | 0.418 | 0.426 | 0.623 | 0.486 |
| m22 | σ21 | 0.7 | 5 | 0.501 | 0.506 | 0.5 | 0.11 | 0.536 | 0.537 | 0.533 | 0.503 |
| m22 | σ22 | 0.7 | 5 | 0.668 | 0.604 | 0.722 | 0.177 | 0.48 | 0.49 | 0.682 | 0.53 |
| m22 | σ21 | 0.5 | 5 | 0.626 | 0.621 | 0.594 | 0.108 | 0.608 | 0.602 | 0.6 | 0.628 |
| m22 | σ22 | 0.5 | 5 | 0.754 | 0.663 | 0.809 | 0.172 | 0.548 | 0.562 | 0.794 | 0.593 |
| m22 | σ21 | 0 | 5 | 0.981 | 0.984 | 0.83 | 0.194 | 0.926 | 0.931 | 0.892 | 0.993 |
| m22 | σ22 | 0 | 5 | 1 | 0.996 | 0.969 | 0.232 | 0.848 | 0.86 | 0.985 | 0.98 |
| m23 | σ21 | 0.9 | 5 | 0.09 | 0.082 | 0.109 | 0.129 | 0.099 | 0.102 | 0.095 | 0.087 |
| m23 | σ21 | 0.7 | 5 | 0.081 | 0.074 | 0.104 | 0.076 | 0.106 | 0.113 | 0.104 | 0.077 |
| m23 | σ21 | 0.5 | 5 | 0.083 | 0.071 | 0.1 | 0.067 | 0.15 | 0.154 | 0.141 | 0.076 |
| m23 | σ21 | 0 | 5 | 0.165 | 0.119 | 0.18 | 0.083 | 0.179 | 0.185 | 0.15 | 0.129 |
| m21 | σ21 | 0.9 | 10 | 0.301 | 0.299 | 0.301 | 0.145 | 0.314 | 0.315 | 0.317 | 0.302 |
| m21 | σ22 | 0.9 | 10 | 0.37 | 0.357 | 0.407 | 0.808 | 0.271 | 0.273 | 0.415 | 0.298 |
| m21 | σ21 | 0.7 | 10 | 0.361 | 0.364 | 0.337 | 0.155 | 0.361 | 0.361 | 0.363 | 0.364 |
| m21 | σ22 | 0.7 | 10 | 0.453 | 0.414 | 0.507 | 0.476 | 0.277 | 0.278 | 0.449 | 0.361 |
| m21 | σ21 | 0.5 | 10 | 0.407 | 0.403 | 0.394 | 0.168 | 0.4 | 0.402 | 0.395 | 0.407 |
| m21 | σ22 | 0.5 | 10 | 0.543 | 0.48 | 0.584 | 0.374 | 0.336 | 0.341 | 0.554 | 0.411 |
| m21 | σ21 | 0 | 10 | 0.881 | 0.917 | 0.644 | 0.469 | 0.689 | 0.691 | 0.655 | 0.925 |
| m21 | σ22 | 0 | 10 | 0.952 | 0.957 | 0.835 | 0.699 | 0.559 | 0.566 | 0.865 | 0.883 |
| m22 | σ21 | 0.9 | 10 | 0.721 | 0.723 | 0.723 | 0.3 | 0.717 | 0.714 | 0.715 | 0.721 |
| m22 | σ22 | 0.9 | 10 | 0.894 | 0.765 | 0.912 | 0.989 | 0.669 | 0.678 | 0.914 | 0.716 |
| m22 | σ21 | 0.7 | 10 | 0.817 | 0.817 | 0.811 | 0.368 | 0.801 | 0.801 | 0.799 | 0.822 |
| m22 | σ22 | 0.7 | 10 | 0.934 | 0.83 | 0.957 | 0.985 | 0.788 | 0.797 | 0.965 | 0.801 |
| m22 | σ21 | 0.5 | 10 | 0.882 | 0.883 | 0.857 | 0.444 | 0.891 | 0.889 | 0.878 | 0.887 |
| m22 | σ22 | 0.5 | 10 | 0.975 | 0.901 | 0.989 | 0.954 | 0.857 | 0.864 | 0.986 | 0.883 |
| m22 | σ21 | 0 | 10 | 1 | 1 | 0.989 | 0.972 | 0.999 | 0.999 | 1 | 1 |
| m22 | σ22 | 0 | 10 | 1 | 1 | 1 | 1 | 0.998 | 0.998 | 1 | 1 |
| m23 | σ21 | 0.9 | 10 | 0.114 | 0.081 | 0.128 | 0.616 | 0.128 | 0.132 | 0.124 | 0.096 |
| m23 | σ21 | 0.7 | 10 | 0.117 | 0.076 | 0.151 | 0.41 | 0.136 | 0.143 | 0.124 | 0.096 |
| m23 | σ21 | 0.5 | 10 | 0.114 | 0.075 | 0.158 | 0.288 | 0.196 | 0.206 | 0.194 | 0.094 |
| m23 | σ21 | 0 | 10 | 0.278 | 0.168 | 0.324 | 0.235 | 0.321 | 0.322 | 0.298 | 0.191 |
