# Supplementary material for: A Comparison of Aggregate P-Value Methods and Multivariate Statistics for Self-Contained Tests of Metabolic Pathway Analysis
Source: PLoS One. 2015 Apr 30;10(4):e0125081. doi: 10.1371/journal.pone.0125081 (PMC4415974; doi:10.1371/journal.pone.0125081)
Supplement: S6 Table — (DOCX) [file pone.0125081.s008.docx]

S_Table 6: Two-sided t-tests, 4 variables, large samples: Fisher vs. Srivastava-Du

| MU | σ | ρ | N | FP | SD |
| --- | --- | --- | --- | --- | --- |
| m11 | σ11 | 0.9 | 20 | 0.355 | 0.354 |
| m11 | σ12 | 0.9 | 20 | 0.506 | 0.533 |
| m11 | σ11 | 0.7 | 20 | 0.4 | 0.399 |
| m11 | σ12 | 0.7 | 20 | 0.582 | 0.612 |
| m11 | σ11 | 0.5 | 20 | 0.508 | 0.498 |
| m11 | σ12 | 0.5 | 20 | 0.711 | 0.737 |
| m11 | σ11 | 0 | 20 | 0.734 | 0.712 |
| m11 | σ12 | 0 | 20 | 0.907 | 0.909 |
| m12 | σ11 | 0.9 | 20 | 0.896 | 0.895 |
| m12 | σ12 | 0.9 | 20 | 0.986 | 0.995 |
| m12 | σ11 | 0.7 | 20 | 0.919 | 0.918 |
| m12 | σ12 | 0.7 | 20 | 0.996 | 0.997 |
| m12 | σ11 | 0.5 | 20 | 0.976 | 0.973 |
| m12 | σ12 | 0.5 | 20 | 0.999 | 0.999 |
| m12 | σ11 | 0 | 20 | 1 | 1 |
| m12 | σ12 | 0 | 20 | 1 | 1 |
| m11 | σ11 | 0.9 | 50 | 0.741 | 0.742 |
| m11 | σ12 | 0.9 | 50 | 0.915 | 0.933 |
| m11 | σ11 | 0.7 | 50 | 0.826 | 0.822 |
| m11 | σ12 | 0.7 | 50 | 0.957 | 0.964 |
| m11 | σ11 | 0.5 | 50 | 0.869 | 0.862 |
| m11 | σ12 | 0.5 | 50 | 0.983 | 0.983 |
| m11 | σ11 | 0 | 50 | 0.989 | 0.99 |
| m11 | σ12 | 0 | 50 | 1 | 1 |
| m12 | σ11 | 0.9 | 50 | 1 | 1 |
| m12 | σ12 | 0.9 | 50 | 1 | 1 |
| m12 | σ11 | 0.7 | 50 | 1 | 1 |
| m12 | σ12 | 0.7 | 50 | 1 | 1 |
| m12 | σ11 | 0.5 | 50 | 1 | 1 |
| m12 | σ12 | 0.5 | 50 | 1 | 1 |
| m12 | σ11 | 0 | 50 | 1 | 1 |
| m12 | σ12 | 0 | 50 | 1 | 1 |
