# Supplementary material for: A Comparison of Aggregate P-Value Methods and Multivariate Statistics for Self-Contained Tests of Metabolic Pathway Analysis
Source: PLoS One. 2015 Apr 30;10(4):e0125081. doi: 10.1371/journal.pone.0125081 (PMC4415974; doi:10.1371/journal.pone.0125081)
Supplement: S7 Table — (DOCX) [file pone.0125081.s009.docx]

S Table 7: Pathway code from human metabolomics study

| CODE | PATHWAY NAME |
| --- | --- |
| P01 | Alanine and Aspartate Metabolism |
| P02 | Benzoate Metabolism |
| P03 | Carnitine Metabolism |
| P04 | Creatine Metabolism |
| P05 | Fatty Acid Metabolism (also BCAA Metabolism) |
| P06 | Fatty Acid Metabolism(Acyl Carnitine) |
| P07 | Fatty Acid, Dicarboxylate |
| P08 | Fatty Acid, Monohydroxy |
| P09 | Food Component/Plant |
| P10 | Fructose, Mannose and Galactose Metabolism |
| P11 | Gamma-glutamyl Amino Acid |
| P12 | Glutamate Metabolism |
| P13 | Glycerolipid Metabolism |
| P14 | Glycine, Serine and Threonine Metabolism |
| P15 | Glycolysis, Gluconeogenesis, and Pyruvate Metabolism |
| P16 | Hemoglobin and Porphyrin Metabolism |
| P17 | Leucine, Isoleucine and Valine Metabolism |
| P18 | Long Chain Fatty Acid |
| P19 | Lysine Metabolism |
| P20 | Lysolipid |
| P21 | Medium Chain Fatty Acid |
| P22 | Methionine, Cysteine, SAM and Taurine Metabolism |
| P23 | Monoacylglycerol |
| P24 | Nicotinate and Nicotinamide Metabolism |
| P25 | Phenylalanine and Tyrosine Metabolism |
| P26 | Phospholipid Metabolism |
| P27 | Polypeptide |
| P28 | Polyunsaturated Fatty Acid (n3 and n6) |
| P29 | Primary Bile Acid Metabolism |
| P30 | Purine Metabolism, (Hypo)Xanthine/Inosine containing |
| P31 | Purine Metabolism, Adenine containing |
| P32 | Pyrimidine Metabolism, Uracil containing |
| P33 | Secondary Bile Acid Metabolism |
| P34 | Steroid |
| P35 | Sterol |
| P36 | TCA Cycle |
| P37 | Tryptophan Metabolism |
| P38 | Urea cycle; Arginine and Proline Metabolism |
| P39 | Xanthine Metabolism |
